# Fine Scale Patterns of Population Structure and Connectivity in Scandinavian Flat Oysters in Scandinavia ( *Ostrea edulis* L.)

**DOI:** 10.1111/eva.70096

**Published:** 2025-03-31

**Authors:** Chloé Robert, Homère J. Alves Monteiro, Alan Le Moan, Arnaud Tanguy, Ane T. Laugen, Jakob Hemmer‐Hansen, Åsa Strand, Pierre De Wit

**Affiliations:** ^1^ Department of Marine Sciences, Tjärnö Marine Laboratory University of Gothenburg Strömstad Sweden; ^2^ Center for Evolutionary Hologenomics, University of Copenhagen Copenhagen K Denmark; ^3^ National Institute of Aquatic Resources, Technical University of Denmark Silkeborg Denmark; ^4^ UMR 7144 AD2M CNRS‐Sorbonne Université Station Biologique de Roscoff Roscoff France; ^5^ Department of Natural Sciences Centre for Coastal Research, University of Agder Kristiansand Norway; ^6^ Department of Environmental Intelligence IVL Swedish Environmental Research Institute Fiskebäckskil Sweden; ^7^ Department of Biological and Environmental Sciences University of Gothenburg Gothenburg Sweden

**Keywords:** genetic diversity, low‐coverage whole‐genome sequencing, management, natural populations, population structure

## Abstract

Stocks of the European flat oyster, *Ostrea edulis* , have collapsed due to overfishing, habitat destruction, and pathogen outbreaks across most of their distribution range. Nonetheless, as a result of lower exploitation pressure and the absence of pathogens in the most northern part of the range, a large part of the remaining wild population can be found in relatively high densities in Scandinavia, a region in Northern Europe. However, despite recent studies focusing on flat oyster population structure along the European coast, little is known about the population structure of oysters in the Skagerrak marginal sea in Scandinavia, and how it is related to neighbouring regions. This study, therefore, aimed to investigate the population structure of flat oysters in Scandinavia, with a special emphasis on the Skagerrak. We gathered low‐coverage whole‐genome sequencing data from oysters in Sweden, Norway, and Denmark, the three countries that border the Skagerrak. Genetic diversity appeared to be homogeneously distributed over the sampled area in the Skagerrak, while samples collected from the east coast of Denmark and from a location with known historical farming activity on the Norwegian West Coast were genetically distinct from Skagerrak samples. A genetic barrier analysis indicated barriers to gene flow in the Baltic Sea transition zone and on the west coast of Norway. Overall, our results suggest that flat oysters from the Swedish Skagerrak coasts form a single panmictic population that is distinct from neighbouring seas, potentially allowing for regional management of stocks and restoration translocations in the area. However, the genetic composition of donor and recipient stocks should be assessed on a case‐by‐case basis, genetic diversity effects of hatchery practices should be monitored, and biosecurity measures need to be considered prior to any movement of stock.

## 1 Introduction

The knowledge of how natural populations are spatially distributed and how they are inter‐connected is a key step in defining management units (Gagnaire et al. 2015; Jansson et al. 2023). The field of population genetics offers a powerful toolbox to describe such units by inferring population structure and connectivity patterns from data on shared alleles and differences in allele frequencies among populations (Reiss et al. 2009). Genetic differences between populations can arise neutrally from the balance between genetic drift and migration; while drift increases differences, migration causes homogenisation of allele frequencies between populations (Wright 1940). Differentiation between populations can arise when physical barriers limit the dispersal of organisms or when changes in the environment favour different alleles in distinct geographic areas by natural selection (Yeaman and Whitlock 2011). The existence of barriers can, when known, be integrated into management plans to avoid mismatches between biological and management units. This knowledge contributes to the sustainable management of biological resources, limiting the loss of genetic diversity and the decline of populations Reiss et al. (2009).

Marine species are often characterised by large effective population sizes (Ward et al. 1994), such that even low migration rates are sufficient to prevent genetic drift from building up genetic differences. This reduces the ability to detect dispersal barriers. Previous work using population genetics with a limited number of genetic markers often reports cases of genetic panmixia over large geographical scales (e.g., Smith et al. 1990; Hedgecock 1994). However, the improvement of DNA sequencing techniques, with an increased number of genetic markers, from tens to millions, has increased the statistical power of the analyses and subsequently revealed the presence of highly structured populations in the sea. It is now possible to detect genomically localised signatures of local adaptation and identify population subdivisions even in cases of low genetic divergence between populations, revealing or rejecting the presence of locally adapted stocks and barriers to gene flow relevant for species management. For instance, in Atlantic cod, population genomic studies have revealed a complex subdivision of the species into two ecotypes: a stationary coastal ecotype and an offshore migratory ecotype (Pampoulie et al. 2023). These ecotypes are often found in different habitats but were historically managed as one unit, which caused depletion of the ecotype with the smallest population size (i.e., the coastal ecotype; Norderhaug et al. 2024). Similar case studies can also be found in other marine species such as European anchovy ( *Engraulis encrasicolus* ; Le Moan et al. 2016), common cockle ( *Cerastoderma edule* ; Vera et al. 2023), vase tunicate ( *Ciona intestinalis* ; Johannesson et al. 2018), spiny lobster ( *Panulirus ornatus* ; Farhadi et al. 2022), and Baltic isopod ( *Idotea balthica* ; De Wit et al. 2020).

European flat oysters, *Ostrea edulis* , are native to European waters. Besides the potential commercial and cultural importance of this species (oysters have been a staple diet of coastal Europeans for millennia, e.g. Botta et al. 2020), flat oysters have an important ecological role in the marine ecosystem. Oyster reefs, formed by living and dead bivalves, create shelter for other marine organisms, and through filtration, they improve water quality and clarity and participate in denitrification (Preston et al. 2020). Overfishing, practised at least since the 19th century, has caused depletion of stocks, and industrial harvesting methods have contributed to habitat destruction (Beck et al. 2011; Zu Ermgassen et al. 2024). Moreover, translocating individuals for aquaculture (Bromley et al. 2016) has introduced parasites such as *Bonamia ostreae* and *Marteilia refringens*, further decimating the remaining stocks (Laing et al. 2006). Consequently, biosecurity has become a major priority in translocation practices (Pouvreau et al. 2023; Zu Ermgassen et al. 2020). Other threats, including increased pollution and competition for resources with invasive species (Colsoul et al. 2021; Nielsen et al. 2017), have also affected the health of flat oyster populations in the past 50 years. In concert, these multiple anthropogenic impacts have caused the decline of the species across the European continent (Zu Ermgassen et al. 2024). In this context, the Swedish flat oyster population is unique, having been mostly protected from the threats affecting other European stocks. Swedish law protects wild oysters, giving the sole control of harvesting to the landowners (SFS 1993:787). This has resulted in a relatively well‐controlled fishing effort over time, with translocations usually being rare and, when performed, well documented. This has also prevented the introduction of harmful parasites (Swedish Board of Agriculture 2022; Mortensen et al. 2023), thus maintaining a relatively large stock size compared to other European populations (Thorngren et al. 2019). Consequently, Sweden currently harbors one of the highest natural densities of flat oysters in Europe.

Due to its ecological and commercial values, there is a European consensus towards the importance of restoring the flat oyster in its native range (Pogoda et al. 2019), using a variety of methods, including large‐scale re‐laying of hatchery‐bred oysters and translocation of adult individuals as broodstock (Zu Ermgassen et al. 2021). These restoration efforts benefit from in‐depth knowledge about the genetic structure of the current stocks (Colsoul et al. 2021; Zu Ermgassen et al. 2020). Understanding the genetic structure and the levels of diversity of stocks is crucial to the establishment of efficient conservation measures, particularly when translocations are envisaged (Pouvreau et al. 2023). Overlooking genetic diversity prior to restoration can lead to maladaptation by replacing locally adapted alleles with non‐adapted alleles, hence reducing the fitness of recipient populations (Laikre et al. 2010). Additionally, the offspring of parents from separate genetic populations could present a reduced fitness, a phenomenon known as outbreeding depression. On the contrary, when the genetic makeup of source and recipient populations is known, this information can be used to limit negative genetic effects to natural populations introduced through translocation and restoration activities. In addition, genomic data can provide detailed information about climate change vulnerability and inbreeding, which can be used in genetic and evolutionary rescue conservation genetic approaches (Hoffman et al. 2020; Whiteley et al. 2015). Whole genome sequencing methods can now provide much more information about the genetic status in restoration compared to earlier studies on flat oysters which were, as for other species, limited to a few genetic markers (i.e., Blanc et al. 1986; Saavedra et al. 1995; Launey et al. 2002; Diaz‐Almela et al. 2004).

Recently, the publication of four reference genome sequences within 2 years (Adkins et al. 2023; Boutet et al. 2022; Gundappa et al. 2022; Li et al. 2023) has made genome‐wide studies possible (i.e., Alves Monteiro et al. 2024; Sambade et al. 2022). These studies have concluded that flat oysters exhibit a pattern of isolation by distance in the wild on a European scale (thousands of kilometres), despite a high potential dispersal rate mediated by a planktonic larval stage, allowing for transport with oceanic currents for several weeks before settling (Buroker 1985). In the Scandinavian waters of the Kattegat and Skagerrak, between the North Sea and the Baltic, the population genetics of oysters has, however, not been well studied. This is a critical knowledge gap given the relative size of the Swedish oyster population in a European restoration and oyster management perspective (Thorngren et al. 2019). An allozyme study from 1989 by Johannesson et al. found low levels of genetic diversity in Sweden and Norway. Alves Monteiro et al. (2024) recently analyzed whole genome sequencing data from oysters collected at 32 different sites across Europe but included only a limited number of sampled sites from the Kattegat and Skagerrak along the Swedish coastline. Seven distinct genetic clusters were identified, separating populations from Skagerrak, the Atlantic Ocean, and the Mediterranean Sea (Alves Monteiro et al. 2024). Although patterns of structure were identified on the Norwegian West Coast, the low numbers of sites prevented detailed investigations of structure within the Kattegat–Skagerrak area, the water body on the Swedish West Coast. The documented translocations in Europe between Denmark, Norway, and other countries (Bromley et al. 2016; Fomsgaard and Petersen 2015; Mortensen 2017), in combination with the low exploitation and lack of pathogens in Sweden, suggest that flat oysters in Skagerrak and Kattegat may harbor genetic diversity that has been lost in other areas. Nonetheless, Scandinavian oysters are also the result of a recent range expansion as they have only colonized the area after the last glacial maximum roughly 13,000 years ago (Delongueville and Scaillet 2003). Genetic diversity in the area could then be relatively low and homogeneously distributed. Local‐scale genomic analyses are therefore essential to ensure the proper management of flat oyster populations in Scandinavia and to enable sound restoration practices in Europe to maintain genetic diversity of natural populations.

This study therefore aimed to characterise patterns of genetic diversity and population structure of flat oysters along Scandinavian coastlines, in the Skagerrak‐Kattegat region. We hypothesized that Swedish oysters would form a genetically distinct population compared to the two other Scandinavian countries, as flat oysters have been actively translocated between aquaculture facilities in Denmark and Norway, but not Sweden (Mortensen 2017). We thus predicted to find genetic differences between Swedish and Norwegian/Danish oysters. We explored the fine‐scale genetic diversity, connectivity, and population divergence among flat oysters sampled at twenty different localities in Sweden, Norway, and Denmark, using millions of SNP markers identified using low‐coverage whole‐genome sequencing. Through population structure analyses and spatial interpretations of genetic data, we searched for barriers to gene flow along the Scandinavian coastline. Knowledge of the population structure of the flat oyster stocks is essential to identify management units within Kattegat and Skagerrak, as well as for international collaboration on the conservation and restoration of this important keystone species.

## 2 Materials and Methods

### 2.1 Material Collection: Sampling, DNA Extraction and Sequencing

**FIGURE 1:**
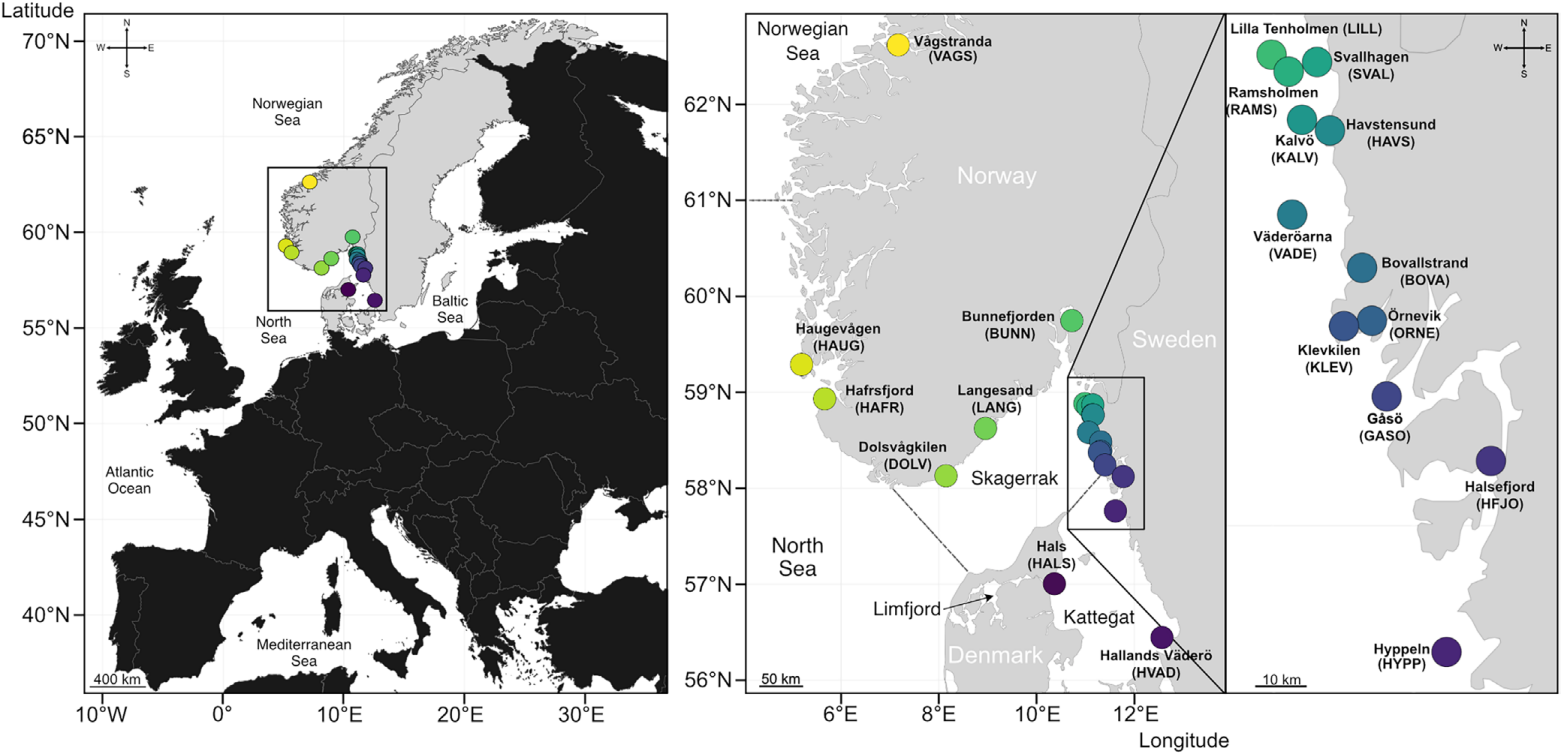
Geographical distribution of the sites included in this study. Flat oysters were collected in Scandinavia (Norway, Sweden and Denmark, countries colored in grey), from a total of 20 sites. Across all sites, a median of 18 oysters were collected in 2020 and 2021, for low‐coverage whole‐genome sequencing analyses. The sites are color‐coded according to coastline location, starting from yellow (central Norway—“VAGS”) to purple (Denmark—“HALS”). Only the water bodies mentioned in this study are annotated on the map.

European flat oysters were sampled in 2020 and 2021 from 13 localities in Sweden, six localities in Norway, and one locality in Denmark (Figure 1; Table S1). Groups of individuals sampled at a specific location at a specific time will be referred to as “samples”. The sampling locations were chosen to provide a representative (20–50 km) sampling design across the entire Skagerrak coast (although limited by the absence of oysters in the southern Skagerrak). Gills and muscle tissues were preserved in 96% ethanol, and DNA was extracted using the DNEasy Blood & Tissue kit (Qiagen), following the standard protocol. Individuals were sequenced using low‐coverage whole‐genome sequencing (lcWGS) at a coverage target of ~1.5× per individual. Randomised library batches of 96 individuals were set up, including two technical replicates per batch. A dual‐indexed library was prepared for each individual using a cost‐effective protocol based on Illumina's Nextera reagents (Therkildsen and Palumbi 2017). The library preparation consisted of four main steps: DNA normalisation, tagmentation, amplification, and PCR clean up with size selection. First, DNA was normalised to a concentration of 2.5 ng/μL. Next, 2.5 μL of DNA extract was fragmented, and adapters were added at the end of each fragment. The fragments were then amplified using PCR and indexed to enable their identification. Finally, residual PCR components were removed, and only DNA fragments of the desired size range were kept (~350–500 bp). The libraries were sequenced with paired‐end 150‐bp reads on an Illumina NovaSeq 6000.

### 2.2 Sequence Filtering and Alignment

**FIGURE 2:**
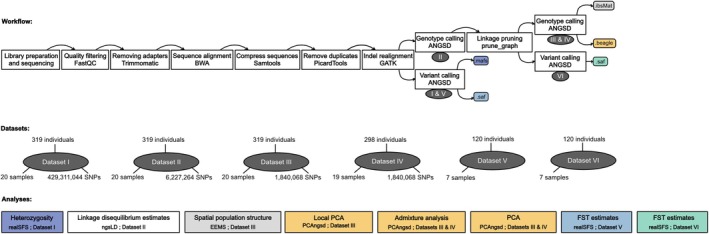
Method workflow, analyses, and datasets included in the present study. *Workflow*: The program used is mentioned for each step. When a particular step creates a dataset later used for an analysis, this dataset is highlighted in a dark oval. The different colors correspond to the different input formats used in the analyses. *Datasets*: Number of individuals and samples, the groups of individuals collected at a specific location and specific time, included in a particular dataset. Samples refer to the number of SNPs specified when applicable. *Analyses*: Program and datasets used for each analysis.

Figure 2 summarises the workflow steps and the analyses carried out in this study and provides relevant information about the datasets and programs used. Sequences were obtained for 364 individual oysters (including 26 technical replicates; Table S2). Sequences were quality filtered (Q > 30) following a quality check using FastQC v. 0.11.8 (Andrews 2010), and adapters were clipped off the ends of reads using Trimmomatic v. 0.39 (Bolger et al. 2014). The sequences were mapped to a chromosome‐level genome assembly (Boutet et al. 2022) using the BWA‐MEM algorithm v. 0.7.17 provided by the Burrows‐Wheeler Alignment tool (BWA) (Li and Durbin 2009). Samtools v. 1.17 (Li et al. 2009) was used to compress the sequences to bam (binary alignment map) format and index them. PCR duplicates were marked and removed from the dataset using the Picard Tools MarkDuplicates v. 2.20.4 (Broad Institute 2019), and GATK v. 4.1.4.1 (McKenna et al. 2010) was used for InDel realignment. The alignment quality and read depth were controlled using Samtools. Technical replicates, closely related individuals, and outlier individuals were removed from the analysis (Figure S3) as is common practice in order to avoid skewing the PCA and admixture analyses. In total, sequences from 319 individuals remained in the analysis, from here on referred to as “*Dataset I*”.

### 2.3 Genotype Likelihood Estimation and Linkage Disequilibrium Pruning

The pipeline followed the low‐coverage whole genome sequencing guide by Lou et al. (2021) (Figure 2). We first analyzed the sequences using ANGSD v. 0.939 (Korneliussen et al. 2014). This software is designed for low‐coverage data using probabilistic estimation of genotypes and allele frequencies. ANGSD was used to identify SNPs across all individuals and to estimate the likelihood of each genotype. We only kept uniquely mapping reads and sites shared by a minimum of 75% of the individuals, which caused a data loss of less than 50% as assessed by the script “plotQC.R” from the ngsTools package v. 1.0.2 (Fumagalli et al. 2014). Reads and bases with a quality below 20 (Phred33 format) were discarded (−minMapQ 20, −minQ 20). Sites with a minor allele frequency below 0.005 were removed from the analysis (−minMaf). Major and minor alleles were inferred from the genotype likelihood (−doMajorMinor 1), and their frequency probability distributions were calculated (−doMaf 1, ‒doPost 1and −doGeno 8). This dataset is from here on referred to as “*Dataset II*”. Next, genomic regions with elevated linkage disequilibrium (LD) were identified and removed from the dataset. The .*pos* file listing the site coordinates and the input file with genotypes .*geno* were used to estimate pairwise LD using ngsLD v. 1.1.1. The estimate of LD was limited to SNPs located at a maximum distance of 50 SNPs from each other (‐‐max_snp_dist 50). This threshold was based on the LD decay plot (Figure S4), as it corresponded to a linkage coefficient of roughly ≤ 0.1. The output of ngsLD consisted of linkage values for each pair of sites included in the filter range. This file was used by the program prune_graph v. 0.3.1 (https://github.com/fgvieira/prune_graph) to prune linked sites from the dataset. More precisely, SNPs with an *r* ^2^ ≥ 0.1 and with a physical distance below 50 kbp were pruned from the dataset (‐‐weight‐filter “column_3 ≤ 50000 && column_7 ≥ 0.1”). ANGSD was then used to estimate the likelihood of each genotype, this time limiting the computation to LD‐pruned sites using the filter ‒sites (from here on referred to as “*Dataset III*”). Using this dataset, ANGSD was run again to estimate an identity‐by‐state matrix using single‐read sampling for LD‐pruned SNPs. The genotyping calling steps were repeated with a reduced dataset, where a genetically divergent sample, “VAGS”, from central Norway, was excluded, following the results of population structure analyses and statistics (see following sections). This reduced dataset will be referred to as “*Dataset IV*” (see Results section). PCAngsd v. 0.982 (Meisner and Albrechtsen 2018) was used to compute a covariance matrix and estimate individual admixture proportions. The software calculated the optimal K and the individual admixture values (q‐values) from the ANGSD beagle output, using *Dataset III* and *Dataset IV*. For a summary of the different datasets, including numbers of individuals and loci see Figure 2.

### 2.4 Population Structure Analyses

The admixture coefficients from PCAngsd were plotted using R v. 4.3.1. (R Core Team 2023), for *Dataset III* and *Dataset IV*. Plotting was done using the function *plotAdmixture* from the R script “admixturePlotting_v5.R” (https://github.com/z0on/2bRAD_denovo) created by Mikhail V Matz. The genetic differentiation among individuals was visualised on a Principal Component Analysis (PCA) using the R package **ggplot2** v. 3.5.1 (Wickham 2016) based on the PCAngsd covariance matrix. The barriers to gene flow were identified using EEMS, “Estimating Effective Migration Surfaces” (Petkova et al. 2016) using *Dataset III*. This software uses a Bayesian MCMC framework to infer genetic diversity differences and impute probable migration routes and barriers on a map, based on the average pairwise genetic distances between the sampled individuals. To optimise the computation, a simple polygon was drawn along the shore, regardless of the presence of islands and the particular topography of the coastline (Figure S5). The identity‐by‐state matrix from the ANGSD LD‐pruned analysis was used by the program runeems_snps, the version of the program adapted for SNP data (https://github.com/dipetkov/eems/tree/master/runeems_snps; commit #ede53b0). Three chains were run simultaneously, for 150 M iterations, with the first 5 M iterations discarded (burn‐in) and 9999 iterations to thin between two writing steps. The R package **rEEMSplots** v. 0.0.2 (https://github.com/dipetkov/reemsplots2; Petkova 2024) was used to visualise the analysis results. The same analysis was performed with several reduced datasets, where one sample had selectively been removed. Removed samples were located on either side of a barrier, in order to visualise the implication of specific samples on the patterns inferred by the EEMS analysis.

### 2.5 Population Genetics Statistics

We computed population genetics estimates using ANGSD v. 0.939 (Korneliussen et al. 2014). The global estimate of heterozygous genotypes was estimated using .mafs files from *Dataset I*. The computation of these files was limited to sites with a minimum sequencing depth of 450 (global value for the dataset, equivalent to a sequencing depth of 1.41× per individual, the targeted coverage value) and a maximum depth of 1500 (equivalent to a sequencing depth of 4.7× per individual, the maximum depth of coverage observed (Figure S6)). The allelic frequency likelihood was then computed for each individual using the option ‒doSaf, restricted to the sites previously filtered. The fraction of heterozygous genotypes was calculated from the folded site frequency spectrum (SFS) computed by the program realSFS in ANGSD. We selected seven samples: four from Norway, “VAGS”; “HAFR”; “DOLV”; “LANG”, two from Sweden, “LILL”; “HFJO”, and one from Denmark, “HALS”, for investigating genome‐wide *F*_ST_ distribution across Scandinavian samples. These samples were chosen because of their geographic spread and their differences in genetic structure, as seen in the results of PCA, admixture, and EEMS analyses using *Dataset III*. All individuals from the selected samples were included in the analysis.

To calculate *F*_ST_, the posterior probabilities of sample allele frequency (SAF) were computed using ANGSD, both for all sites and for LD‐pruned sites, generating *Dataset V* and *Dataset VI*, respectively. The SAF files were then used to compute pairwise folded 2D‐SFS, which were used as input priors for *F*_ST_ calculations according to Bhatia et al. (2013), as recommended by the ANGSD manual. *F*_ST_ was calculated using sliding windows of 100 kb and steps of 50 kb. Data wrangling and plotting were performed using the R packages **dplyr** v. 1.1.3 (Wickham et al. 2023) and **ggplot2** v. 3.5.1 (Wickham 2016). The global *F*_ST_ was also calculated for each sample pair in *Dataset V* and *Dataset VI*.

A Mantel test was performed to test whether genetic differentiation was correlated with geographical distance, which is expected under an Isolation‐by‐Distance (IbD) pattern. The analysis was run both for the entire dataset and for the dataset excluding the most genetically distinct population (Datasets III & IV). The covariance matrices from ANGSD were used as input for genetic distance data between individuals. The geographical distances between each pair of sampled sites were calculated using the R package **geosphere** v. 1.5–20 (Hijmans 2024), by computing the shortest distance between two samples. The Mantel test was run using the function mantel.randtest from the R package **adegenet** v. 2.1.10 (Jombart 2008; Jombart and Ahmed 2011), with 10^6^ permutations. Additionally, a linear regression was also performed for both datasets to further assess the correlation between the genetic and geographic matrices. The result was plotted as a scatterplot with density colouring, following an estimation of kernel density using the function kde2d available in the R package **MASS** v. 7.3–60 (Venables and Ripley 2002).

### 2.6 Local PCA

We performed local PCAs for each scaffold to detect chromosomal rearrangements along the genome following the script “run_local_pca.sh” (https://github.com/therkildsen‐lab/genomic‐data‐analysis/blob/master/scripts/run_local_pca.sh; commit #a003db1) using *Dataset III* as input. Local PCAs were calculated using sliding windows of 1000 SNPs. In each window, a SNP covariance matrix was calculated using PCAngsd, after which an eigen decomposition was performed using two PCs to detect the patterns of genetic variation. The mean SNP loading value of each window was plotted against the mean position of the windows across the genome using the R package **lostruct** v. 0.0.0.9000 (Li and Ralph 2019) and the R script “NIC_Localpca_EUostrea_15feb23.R” (https://github.com/HomereAMK/EUostrea/blob/main/Rscripts/NIC_Localpca_EUostrea_15feb23.R; commit #65321de). Local PCAs allow us to identify large regions of the genome contributing disproportionally to the divergence pattern across the samples, which can be expected from chromosomal rearrangements (e.g., an inversion). Following the identification of such rearranged regions, two local PCA analyses were performed for each scaffold exhibiting this pattern. The first local PCA only included SNPs inside the region of interest, and the second local PCA included SNPs outside of the rearranged region. For this purpose, covariance matrices were generated separately for each SNP dataset. These local PCAs were plotted using R, and in the case of a chromosomal rearrangement, the local PCA would group karyotypes into three separated clusters, corresponding to the two homozygote and the heterozygote states. Finally, the karyotype proportions at each site were calculated from the PCA cluster and were plotted on a map using the R package **mapmixture** v. 1.10 (Jenkins 2024).

## 3 Results

**FIGURE 3:**
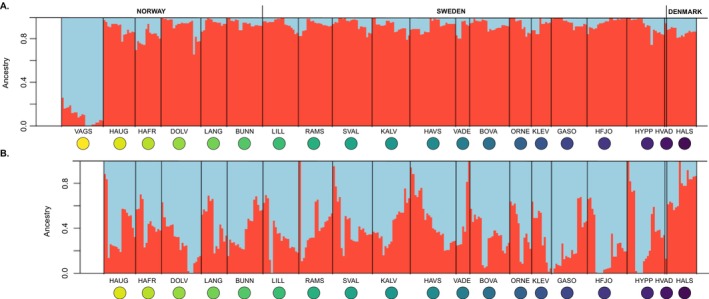
Admixture plots from PCAngsd. Each vertical line represents one individual, and the color shows the proportion of each individual assigned to the *K* = 2 genetic clusters. The labels and colored circles identify the samples. (A) Admixture plot using *Dataset III*, that is, 20 sampling locations and 319 flat oysters; based on 1,840,068 SNPs. (B) Admixture plot using *Dataset IV*, that is, 19 sampling locations and 298 flat oysters; based on 1,840,068 SNPs.

**FIGURE 4:**
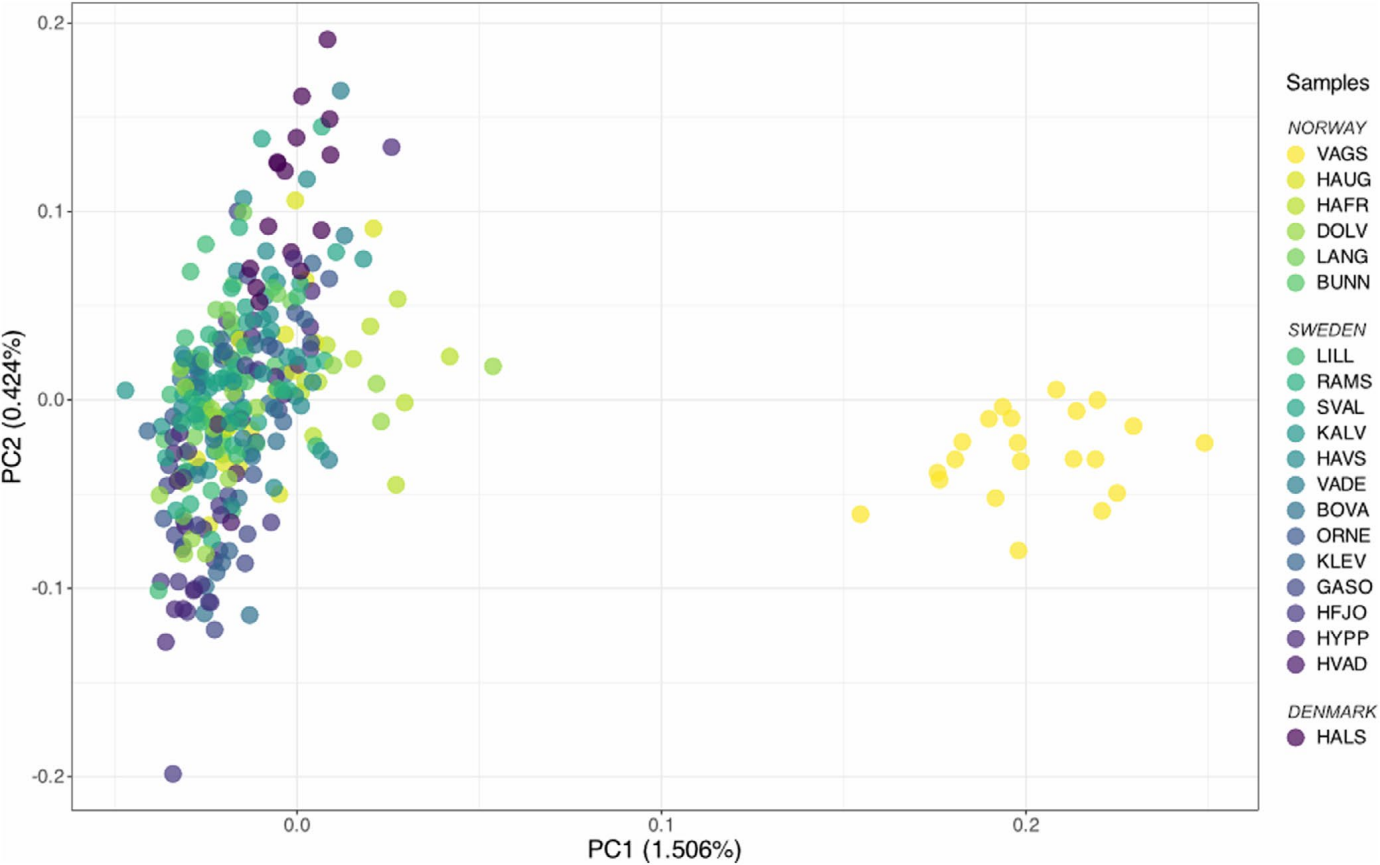
Visualisation of first and second principal components of a principal component analysis (PCA) on 319 flat oysters based on 1,840,068 SNPs (*Dataset III*). Individuals are represented by dots colored by their sample of origin. The colors follow a gradient based on coastline location, starting from yellow (central Norway—“VAGS”) to purple (Denmark—“HALS”). The percentage of variation explained by each principal component is specified on each axis.

From a total of 364 flat oysters collected across the 20 sampling sites in Scandinavia, 319 sequences were kept for further analyses. In total, 6,227,264 SNPs were kept after quality filtering (*Dataset II*), and 1,840,068 SNPs after filtering for physical linkage disequilibrium (*Dataset III*; see Figure 2). The mean coverage was 1.8X across all individuals, and the mean heterozygosity was 0.14 (Figures S6 and S7). The heterozygosity ranged from 0.03–0.29 and was lowest in the Dolsvågkilen, “DOLV”, sample and highest in the sample from Hals, “HALS”, due to high heterozygosity in two individuals (Figure S7). Using the linkage‐pruned dataset, we found no evidence for population structure across the Skagerrak, while the sample from northern Norway (“VAGS”, Vågstranda) was clearly differentiated from the Skagerrak samples (Figures 3 and 4). The Danish sample, “HALS”, was also genetically distinct from the Norwegian and Swedish oysters (Figures S8 and S9).

### 3.1 Population Structure Analyses

The optimal number of clusters (*K*) resulting from the PCAngsd admixture analysis was *K* = 2 both for *Dataset III* (Figure 3A) and *Dataset IV* (Figure 3B). The admixture plot computed with *Dataset III* assigned a distinct ancestry to the flat oysters sampled in “VAGS” in comparison to the individuals sampled in other localities for which a similar ancestry pattern was supported (Figure 3A,S8). Furthermore, two genetically distinct clusters were observed on the PCA (Figure 4). These clusters were separated on PC1, which explained 1.51% of the variation observed in the data (Figure S10). One cluster grouped individuals sampled from “VAGS” on the Norwegian West Coast, while the other cluster gathered the remaining individuals. The second PC, which explained 0.42% of the variation in the data, did not separate individuals by site. The remaining principal components were not plotted as the percentage of variance explained was low (Figure S10). The presence of two clusters on the PCA and the clear separation of individuals collected in “VAGS” was the strongest genetic signal in our results. However, in a PCA excluding “VAGS” individuals (*Dataset IV*, Figure S8), individuals collected in Hals, located at the entrance of the Limfjord on the east coast of Jutland, Denmark, clustered in a corner of the PCA, showing that this sample was distinct from oysters sampled in Norway and Sweden (Figure S8). The distinctness of the Danish sample could also be observed in the admixture plot computed with *Dataset IV*, where the individuals sampled from Hals had different ancestry proportions (0.75 ± 0.08 for K1; 0.25 ± 0.08 for K2) to the rest of the individuals (0.38 ± 0.12 for K1; 0.62 ± 0.12 for K2) (Figure 3B).

**FIGURE 5:**
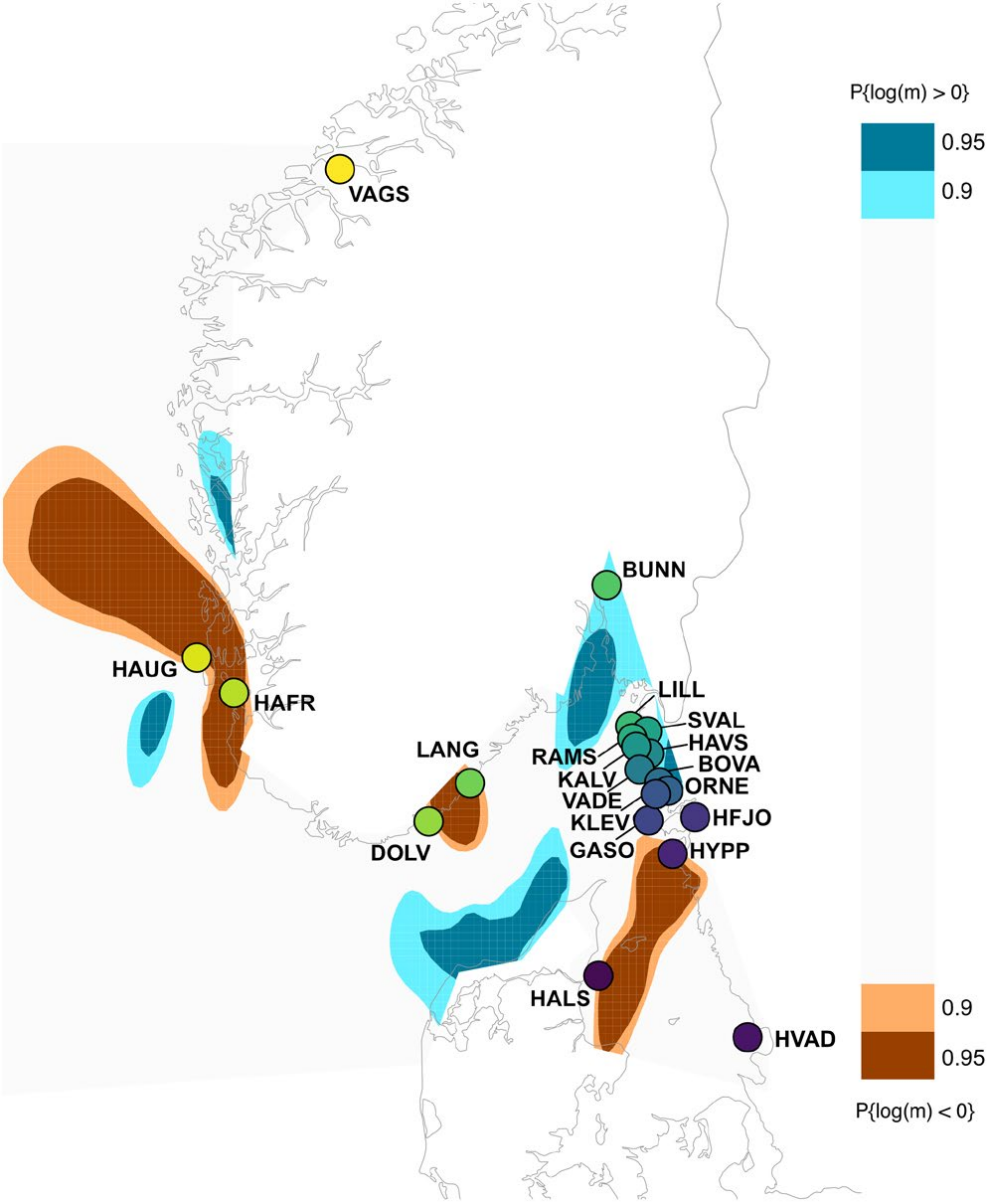
Map showing the effective migration surface, output from the EEMS analysis, computed on 319 flat oysters and based on *Dataset III* (1,840,068 SNPs). Geographic barriers are shown in brown, and regions with high migration rates are shown in blue. The samples included in the analysis are identified with colored dots and acronyms.

Areas with migration barriers (brown) and areas inferred to act as migration corridors (blue) by the EEMS analysis are shown in Figures 5 and S11. Only the areas with posterior probabilities of being corridors or barriers higher than 90% are shown on the map, and the samples included in the analyses are represented by coloured dots. Terrestrial areas of the map should be ignored, as they cannot be considered potential dispersal routes. Three areas limiting migration and genetic exchange were identified in our sampled area (Figure 5). The first one was located in the southern part of the Norwegian West Coast surrounding the locations of Haugevågen, “HAUG”, and Hafrsfjord, “HAFR” and extending to the north. This barrier corresponds to the genetic differentiation between “VAGS” and the other samples, which can also be clearly seen in the PCA and admixture. The second barrier to migration was found between the Gothenburg archipelago and the Danish West Coast, at the border between Skagerrak and Kattegat. This barrier corresponds to the unique genetic composition of the Danish “HALS” sample at the mouth of the Limfjord, also observed in the admixture and PCA results. The last region of restricted migration was situated in the south of Norway, between Dolvågskilen and Langesand, two localities geographically close to each other. This barrier could not clearly be observed in the admixture or PCA results and is only observed when the sample from Langesand “LANG” is included in the dataset (Figure S11). In contrast, the two other barriers, however, were inferred in the same locations in all EEMS runs. Higher gene flow than expected was inferred in Skagerrak, specifically in the area between the Oslo fjord and the northern parts of Denmark. This latter pattern may be an artefact, as no samples were collected in the area.

### 3.2 Population Genetic Statistics

The global *F*_ST_ of *Dataset V* was low, ranging from 0 to 0.01 (Table S12). The global *F*_ST_ was higher for sample pairs including “VAGS”, ranging from 0.036 to 0.056. The global *F*_ST_ was overall lower for *Dataset VI*, the LD‐pruned dataset, as *F*_ST_ ranged from 0 to 0.008 for sample pairs excluding “VAGS”. The range increased for pairwise *F*_ST_ calculation, including “VAGS” ranging from 0.022 to 0.034. The heterozygosity estimates were, however, strongly correlated with sequencing depth in all samples (Figure S13), as shown by the low coverage and low heterozygosity of the “DOLV” sample. This is expected from the type of data analyzed here, limiting our ability to correctly infer heterozygotes at low sequencing depth.

The genome‐wide distribution of *F*_ST_ peaks in the un‐pruned dataset (Figure S14) clearly identified regions of high divergence in many of the pairwise comparisons, in addition to individual outlier loci. In particular, pairwise comparisons involving “VAGS” showed a region at the beginning of scaffold 4 as highly differentiated. After linkage pruning, most of these regions disappeared, although traces of the region on scaffold 4 were still visible to some extent (Figure S15). In contrast, comparisons between Skagerrak samples and “HALS” (Figures S14 and S15) did not show the presence of such localized peaks of differentiation.

The Mantel test for *Dataset III*, including all the samples, showed a correlation (*r* ^2^ = 0.2671, *p* < 0.00001) between genetic and geographic distances (Table S16 and Figure S17). The regression analysis revealed that geographic distance explained 7.1% of the variation in the genetic data (slope = 4.727e‐06, *r* ^2^ = 0.071, *p* < 0.001; Figure S17). Individuals from “VAGS” were genetically more similar to each other than to individuals from other samples. A weaker, yet still significant, correlation was observed when “VAGS” was excluded, using *Dataset IV* (Mantel test: *r* ^2^ = 0.1, *p* < 0.001, Table S16 and Figure S17; linear regression: slope = 1.795e‐06, *r* ^2^ = 0.01, *p* < 0.001).

### 3.3 Local PCA

**FIGURE 6:**
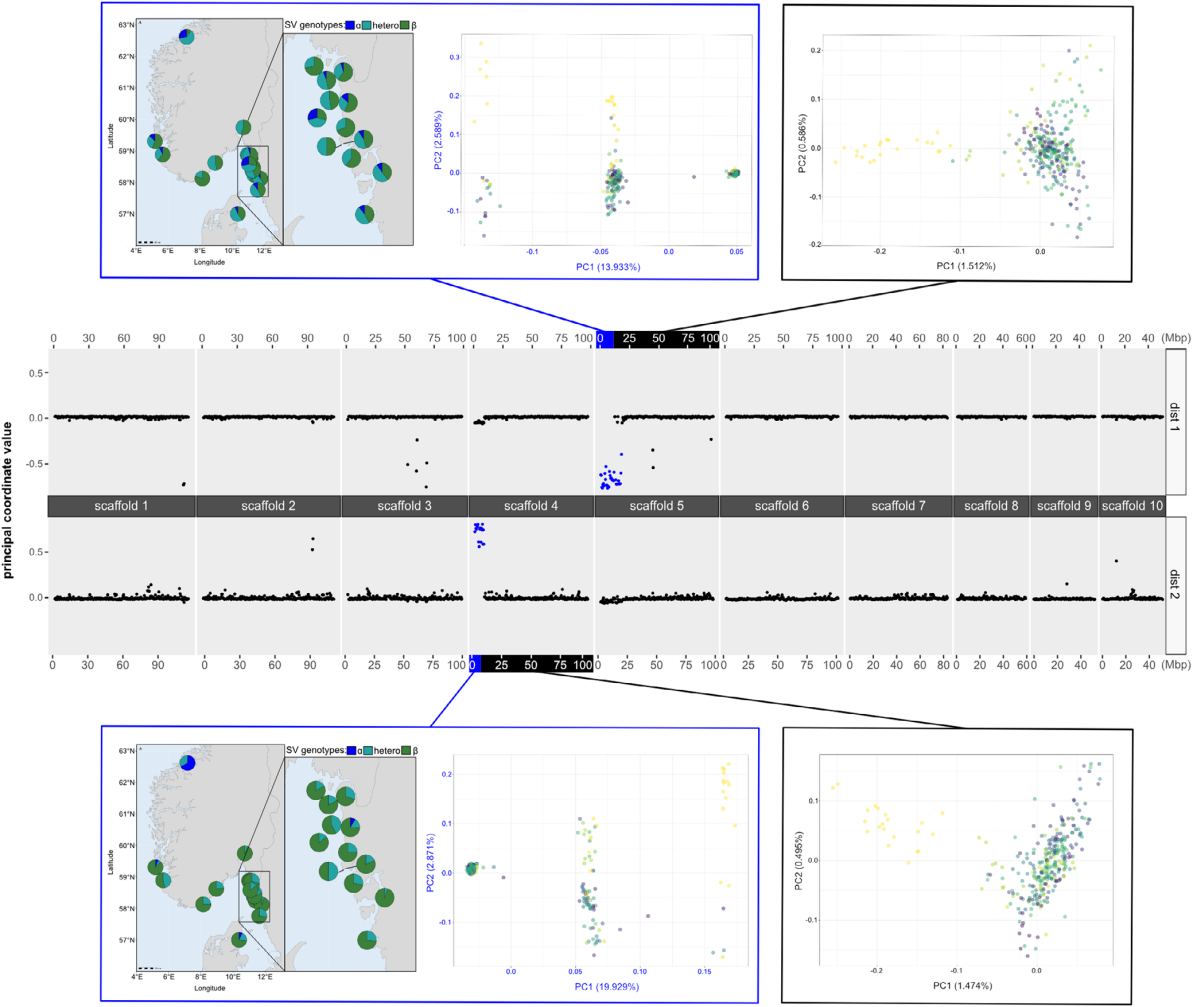
Local PCA on a window size of 1000 SNPS for each scaffold. The first two dimensions of an MDS on the eigen vector loadings are shown, and the structural variants are highlighted in color. PCAs on the SNPs inside and outside of the inversion are shown together with the karyotype proportions of the different structural variant genotypes per sample.

Two strong signals of chromosomal rearrangements were found along the genome, in scaffold 4, start and end positions 14,778–8,040,476 and scaffold 5, start and end positions 299–17,658,073, although in both cases it seemed that the rearrangement reached the end of the chromosome (Figure 6). These structural variants (SV) were designated in a previous study as Chr04:22Kb_sv and Chr05:172Kb_sv respectively (Alves Monteiro et al. 2024).

The PCA plots showed three distinct clusters inside these regions of interest (the right plots inside the blue rectangles on Figure 6), with one cluster located at mid distance from the two others. These clusters were separated on PC1, which explained 19.3% and 13.9% of the variation in the data for scaffold 4 and 5, respectively. Here, samples from the central cluster correspond to the heterokaryotype carrying both hypothesized standard and rearranged parts of a given chromosomal rearrangement, “ɑ/β”, while samples from the two most distant clusters are homokaryotype samples carrying two standard or two rearranged alleles, “ɑ/ɑ” and “β/β”, respectively. In contrast, PCAs on positions outside the SV showed the same differentiation pattern as the genome‐wide average (the plots inside the black rectangles on Figure 6).

The mapmixture maps (the left plots inside the blue rectangles on Figure 6) show that both chromosomal rearrangements are polymorphic in each studied site, but also demonstrate different karyotype proportions for “VAGS” in comparison to other Scandinavian samples for scaffold 4. Most oysters from this sample are homozygous for the “ɑ” arrangement on this scaffold. This karyotype is only present in a few other samples (“HAUG”, the sample from Havstensund “HAVS” and “HALS”), but in smaller proportions. The most common karyotype in all other samples is a “β” homozygote genotype. Similarly, oysters homozygous for the “ɑ” arrangement on scaffold 5 are present in “VAGS”, yet in smaller proportion (28.6%). This karyotype is found in more than half of the other Scandinavian samples, sometimes in a similar proportion as in “VAGS” (e.g., in the sample from Väderöarna “VADE”).

## 4 Discussion

Our data show that the flat oyster population in Skagerrak is overall genetically homogeneous; however, the northernmost sample, which was collected in Norway, Vågstranda, “VAGS” was genetically differentiated from Skagerrak samples, as observed from multiple genetic analyses. In addition, the Danish sample collected at Hals, “HALS” (eastern Jutland) in the Kattegat showed signs of being distinct from others, both by individuals clustering together in a PCA (Figure S8) and by consisting of different ancestry proportions than other samples in an admixture analysis (Figure 3B). Even though PCA analyses have limitations (Lever et al. 2017; McVean 2009; Yi and Latch 2022), the consistency of results between the PCA and the admixture analyses supports the reliability of the identified patterns of population structure. The observed absence of strong population structure throughout Skagerrak is surprising, as this species is strongly marked by isolation by distance (IbD) on a European wide scale (Alves Monteiro et al. 2024; Lapègue et al. 2022; Vera et al. 2016). Although the spatial scale studied here was much smaller than previous European‐wide studies, we still expected some genetic structure given the complex topography of the area, characterized by the presence of thousands of islands along the shore and the presence of important ecological gradients, for example, from fully marine waters to the brackish waters of the Baltic Sea. On the Scandinavian scale, the IbD pattern appears to be strongly driven by the presence of the VAGS sample—without this sample, a weak IbD signal remains due to the high power of the analysis, but geographic distance explains only 1% of the genetic distance.

### 4.1 Lack of Genetic Structure in the Skagerrak

At the onset of this work, we hypothesised that environmental gradients and/or physical dispersal barriers could prevent gene flow between sampling locations, as observed in several other species, including blue mussels ( *Mytilus edulis* ; Gustafsson et al. 2024), eelpout ( *Zoarces viviparus* ; Christiansen and Frydenberg 1974), salmon ( *Salmo salar* ; Nilsson et al. 2001) common green branched weed ( *Cladophora rupestris* ; Johansson et al. 2003), European plaice ( *Pleuronectes platessa* , Le Moan et al. 2021) and many other species in the area (reviewed in Henriksson et al. 2024, Johannesson et al. 2020). However, the lack of inferred barriers in the eastern part of the Skagerrak suggests an overall high connectivity among oysters in the area. The connectivity among flat oyster stocks in different areas might be facilitated by water currents and the duration of the larval stages.

The oysters located in the Bunnefjord, “BUNN”, seem connected to the remaining Scandinavian oysters, which is surprising given their location, far into the south‐eastern end of the Oslo fjord. Hypothetically, the “BUNN” sample could be a “sink” for larvae drifting from southern Skagerrak. Additionally, the oysters could as well have been translocated to the fjord from another source population. Even though translocations have not been documented in the Skagerrak, it is possible that individuals have been moved within the region, particularly given Norway's historical farming activity (Bromley et al. 2016; Johannesson et al. 1989; Zu Ermgassen et al. 2024). It is important to highlight that in the case of a translocation, EEMS interprets the movement of stock as a long‐distance migration event. Without background information, human‐mediated transport cannot be differentiated from other processes (Silliman 2019). Background knowledge on the movement of stocks is hence crucial to correctly interpret the results of EEMS analyses and identify artefacts from real migration.

The observed genetic patterns also can be influenced by historical events, particularly those related to past climate changes, rather than just current environmental conditions and human activities. During the Last Glacial Maximum, Scandinavia was covered by an ice sheet (Gyllencreuzt 2005), and marine species only recolonised the region about 10,000 years ago, after surviving in ice‐free refugia. The limited genetic structure observed in flat oysters in the Skagerrak might therefore be explained by a relatively recent recolonization, likely from a single source population. This hypothesis is supported by previous genetic studies showing that UK oysters are closely related to North European populations, suggesting they share a common ancestral population (Alves Monteiro et al. 2024; Vera et al. 2016). This limited genetic structure and a simple colonization history contrast with genetic patterns in some other species, which show more complex patterns of genetic structure in the Skagerrak, in some cases suggested to result from multiple recolonization events from different source populations (e.g., Gustafsson et al. 2024 and Leder et al. 2021; see also review by Johannesson et al. 2020).

### 4.2 Patterns of Genetic Structure in Scandinavia

#### 4.2.1 Vågstranda Sample: Genetically Distinct

Previous studies have found that structural variants (SV) can play a large role in observed differentiation in flat oysters, especially at the range edges of their European distribution (Alves Monteiro et al. 2024; Lapègue et al. 2022). Such a signal of genetic differentiation was also observed in our analyses, both in high *F*_ST_ levels detected in a large chromosomal region located at the beginning of scaffold 4, Chr04:22Kb_sv, in pairwise comparisons with “VAGS” and in a local PCA (Figures 6 and S14). Linkage pruning reduced the signal of this region by filtering out many linked SNPs. However, the genetic differentiation was not restricted to the SV but was found genome‐wide (Figure 6). We also observed a signal of the SV Chr05:172Kb_sv on scaffold 5 but with a less pronounced geographical pattern (Figure 6). As a result, the separation of “VAGS”, located in central Norway, from all other samples emerged as the strongest pattern in our results. This was supported by the PCA, the admixture analysis, and the EEMS barrier analysis (Figures 3A, 4 and 5).

The genetic differentiation of flat oysters from Vågstranda, the collecting location of the “VAGS” sample, may reflect natural population structure in the species. However, both imports and exports of oysters have also been reported (Mortensen 2017). Individuals have been translocated from the Netherlands and several locations in Norway (Gulen and Sogn, located south of Vågstranda), and hence the effects of past translocations may also be possible. The south‐western tip of Norway, near the city of Egersund, has been shown to form a multi‐species phylogeographic boundary (Blanco Gonzalez et al. 2016). This boundary does, however, not coincide with the pattern that we observe here, as two of our samples north of this boundary on the Norwegian coast (collected in Haugevågen, “HAUG”, and in Hafrsfjord, “HAFR”) were indistinguishable from Skagerrak samples on the PCA and admixture plots.

#### 4.2.2 Border Between Kattegat and Skagerrak

The border between Kattegat and Skagerrak (marked with a dotted line in Figure 1) is a well‐known area for strong genetic differentiation for many marine organisms (Johannesson et al. 2020). This area was also identified as a location of flat oyster population division when “VAGS” was excluded from the PCA, admixture, and EEMS analyses (Figure 3B; Figure S8). Several factors, which are non‐mutually exclusive, can contribute to this pattern, including founder effects, historical isolation with recent secondary contact, local adaptation to low salinity, larval dispersal, water currents, and the introduction of genetic material from the Limfjord.

Founder effects may occur in range‐margin populations in areas that have been colonized after the last glaciation, and/or by divergent selection pressures imposed by the strong environmental gradients in the area. Salinity, in particular, may be a significant factor limiting the population range in Skagerrak and Kattegat, ranging from 30 psu (practical salinity unit) in Skagerrak to 20 psu in Kattegat and 10 psu in the Öresund. In salinity levels lower than 20 psu, settlements of flat oyster larvae are rare, and growth is limited (Davis and Ansell 1962). Limitations in larval dispersal might also play an important role in the observed pattern. Flat oyster females brood their offspring for 10 days; then larvae spend from 10 to 15 days in the water column before settling (Gustafsson et al. 2023). Previous simulations of larval dispersal have shown that the primary source of flat oysters on the Swedish coast could be located in the Gothenburg area (assuming that oyster beds are present in the area), with sink populations located further north along the coastline, creating a one‐way barrier between populations in Skagerrak and Kattegat (Gustafsson et al. 2023). The barrier observed in the EEMS results may thus be explained by the Baltic current, which moves north through the Kattegat into the Skagerrak.

Another potential cause for the observed barrier may be the introduction of genetic material from another population. One possibility could be introgression from the “large” population of oysters in the Limfjord, which is genetically differentiated from Skagerrak/Kattegat (Alves Monteiro et al. 2024) and has been subject to translocations (Fomsgaard and Petersen 2015). Another possibility could be introgression from a historically separated population from a different ice‐age refugium. In either case, the relatively high heterozygosity found in the “HALS” sample (Figure S7), which was collected just at the mouth of the Limfjord in the Kattegat, supports some outbreeding (although the heterozygosity might be affected by the sequencing depth, see below; Figure S13). Blue mussels, which share a similar larval biology with oysters, also exhibit a strong genetic gradient in the Kattegat due to the introgression of genetic material from the Baltic Sea (Stuckas et al. 2017). This supports the idea that the flat oysters in Kattegat could be genetically differentiated from Skagerrak, although the lack of further samples on the Swedish Kattegat coast prevents extrapolation of our data to the entire Kattegat Sea. It is also worth noting that at the southernmost collecting site at Hallands Väderö, “HVAD”, only one individual was found during the sampling. To our knowledge, this is the first time that live flat oysters have been documented this far south in the Kattegat (Naturvårdsverket 2010; SLU Artdatabanken 2024). A census survey of flat oysters in the Kattegat is necessary to assess stock size and population dynamics in the area.

#### 4.2.3 Barrier to Gene Flow in the South of Norway

EEMS also identified a barrier along the southern Norwegian coast, between our two collecting locations Dolsvågkilen, “DOLV”, and Langesand, “LANG”, although no differentiation was observed in the PCA and admixture analyses. EEMS has been shown to be more powerful than PCA at picking up signals in the data, which could explain this discrepancy (Petkova et al. 2016). The flat oysters from “DOLV” were in general sequenced at a lower depth (Figure S6), which correlates with a lower heterozygosity (Figure S13). Potentially, this could have impacted the accuracy of the matrix of pairwise genetic distances used for the EEMS analyses, artificially inferring a barrier between “DOLV” and “LANG”. However, the barrier was also observed when “DOLV” was omitted from the EEMS analysis, while when “LANG” was omitted, the barrier was no longer present, indicating that the “LANG” sample was responsible for the observed pattern (Figure S11). A recent study of blue mussel connectivity in the area (Gustafsson et al. 2024) also found a barrier along this coastline, although further to the north (near Kragerø), warranting further studies in the area.

### 4.3 Observations for Biodiversity Management

Based on our data and for conservation actions, it seems reasonable to consider the samples of flat oysters collected in Skagerrak to represent a single genetic cluster, as they share a similar genetic makeup. The Swedish stock of flat oysters has not been extensively monitored to date, but it is estimated that approximately 36 million oysters inhabit the northern part of Skagerrak (Thorngren et al. 2019), making it a significant proportion of all remaining flat oysters worldwide. However, the status of the reefs remains unknown and there is a lack of understanding of the population dynamics (population size, composition and distribution over time) of flat oysters in Sweden (Zu Ermgassen et al. 2024). Therefore, more efforts should be directed towards documenting and exploring local to regional patterns in population distribution and development, and in small‐scale source‐sink dynamics.

## 5 Conclusion

Our initial hypothesis was that flat oysters from Sweden, in Skagerrak and Kattegat, are genetically distinct from those in other parts of Scandinavia due to the absence of aquaculture translocations and heavy exploitation. However, our study found little genetic structure among samples of oysters in the Skagerrak, indicating that they represent a single panmictic population. Given the ecological importance and severe degradation of flat oyster habitats in Europe, efforts to restore flat oyster populations have been identified as a priority (Zu Ermgassen et al. 2024). Previously, no studies have thoroughly investigated the genetic diversity of Swedish flat oysters. The results of this research are therefore crucial for establishing effective conservation and restoration strategies in the Skagerrak. Our findings suggest that wild Skagerrak oysters are suitable for restoration in genetically homogenous locations within the Swedish parts of the Skagerrak. However, given the presence of population structure at local geographical scales in areas bordering the Skagerrak (Kattegat and the Norwegian Sea; Alves Monteiro et al. 2024), the weak isolation by distance pattern, and our limited sampling coverage along the Norwegian Skagerrak coasts, we recommend that restoration projects on the Norwegian Skagerrak coasts and outside of the Skagerrak should be done with caution and only if supported by genetic data from donor and recipient locations.

Given the presence of population structure at local geographical scales, we generally recommend limiting flat oyster translocations between genetically homogeneous locations and sourcing broodstock for hatchery production of oyster seed for restoration from the recipient area when possible. Future work should expand geographical mapping of population structure in the Norwegian Skagerrak and neighbouring areas to the Skagerrak. Additionally, biosecurity measures and consideration of genetic diversity in hatchery‐reared spats are crucial for successful restoration, addressing potential reductions in genetic diversity and inbreeding issues (Alves Monteiro et al. 2022; Pouvreau et al. 2023).

## Conflicts of Interest

The authors declare no conflicts of interest.

## Supporting information


**Table S1.** Details of the sampling localities included in this study. Some locations are new to science, others were previously included in the study by Alves Monteiro et al. (2024).

**Table S2.** Sequencing quality for each individual sequenced.

**Figure S3.** Hierarchical clustering and PCA on a pre‐filtered dataset of flat oyster sequences, including technical replicates and closely related individuals. The yellow rectangles in the hierarchical clustering highlight the sequences that were removed for further analyses. The grey rectangle highlights hatchery‐bred samples that were not included in the study. The group a was removed because of weird clustering, groups b, c, d and e were removed from the dataset because they are composed of closely related individuals.

**Figure S4.** LD‐decay plot along scaffold 4. The red dotted line represents the threshold selected for linkage pruning.

**Figure S5.** Polygon used for the EEMS analysis. The coordinates were obtained using the Polyline tool available on the Keene State College website https://www.keene.edu/campus/maps/tool/.

**Figure S6.** Depth of coverage, number of reads paired and mapped for each sample. One dot represents one individual.

**Figure S7.** Proportions of heterozygous genotypes in each sample, using *Dataset I*. The red dotted line corresponds to the average level of heterozygosity calculated for all samples. One dot represents one individual.

**Figure S8.** Visualisation of first and second principal components of a principal component analysis (PCA) on 298 flat oysters based on 1,840,068 SNPs (*Dataset IV*). Individuals are represented by dots coloured by their sample of origin. The percentage of variation explained by each principal component is specified on each axis.

**Figure S9.** Proportion of each sample assigned to K1, from the PCAngsd admixture analysis. (A) Using *Dataset III*, that is, 20 sampling locations and 319 flat oysters; based on 1,840,068 SNPs. (B) Using *Dataset IV*, that is, 19 sampling locations and 298 flat oysters; based on 1,840,068 SNPs.

**Figure S10.** Scree plot, percentage of variance explained by each principal component, based on the PCAngsd covariance matrix computed for *Dataset III*.

**Figure S11.** Map showing the effective migration surface, output from the EEMS analysis, after removal of a sample from *Dataset III* (1,840,068 SNPs). The following samples were removed for each map: (A) “VAGS”; (B) “DOLV”; (C) “LANG”; (D) “HVAD”; (E) “HALS”. Geographic barriers are shown in brown, and regions with high migrations are shown in blue. Coloured dots represent the samples included in the analysis.

**Table S12.** Pairwise F_ST_ estimates among sampling locations. Above diagonal: weighted F_ST_ estimates for *Dataset V*, comprising 120 individuals from seven samples; below diagonal: estimates for *Dataset VI*, the LD‐pruned version of Dataset V.

**Figure S13.** Linear regression model showing the relationship between heterozygosity and coverage for each individual included in *Dataset I*. Each dot is one individual; the colour represents the sample of origin.

**Figure S14.** Pairwise F_st_ with a window size of 100 kb and a step of 50 kb among samples of flat oysters, using *Dataset V*. The 10 scaffolds are highlighted with different colours.

**Figure S15.** Pairwise F_st_ with a window size of 100 kb and a step of 50 kb among samples of flat oysters, using *Dataset VI*. The 10 scaffolds are highlighted with different colours.

**Table S16.** Mantel tests of genetic vs. geographic distances for all the samples of flat oysters included in this study (Dataset III) and excluding the sample from Vågstranda (VAGS, dataset IV).

**Figure S17.** Analyses of correlation between genetic and geographic data for the entire dataset of flat oysters included in this study (Dataset III; A, B panels) and for the dataset excluding the VAGS sample (Dataset IV; C, D panels). The genetic dataset is the covariance matrix output from ANGSD, and geographic data has been calculated as the shortest distance between two samples. (A, C) Histograms showing the distribution of 100,000 simulated Mantel correlations, the vertical line shows the observed correlation between the matrices of genetic and geographic data. For both datasets, *p* < 0.00001. (B, D) linear regression between geographic and genetic distance. Colours show the change in density of data points. (B) Slope = 4.727e‐06, *r* ^2^ = 0.071, *p* < 0.001, D: Slope = 1.795e‐06, *r* ^2^ = 0.01, *p* < 0.001.

## Data Availability

All raw genetic data produced in this study are available at NCBI, BioProject PRJNA1029395. The SNP covariance matrix used for population genomic inferences is available as supplemental data appended to the manuscript.
