# Supplementary figures and images for: Fine Scale Patterns of Population Structure and Connectivity in Scandinavian Flat Oysters in Scandinavia ( Ostrea edulis L.)
Source: Evol Appl. 2025 Mar 31;18(4):e70096. doi: 10.1111/eva.70096 (PMC11955839; doi:10.1111/eva.70096)

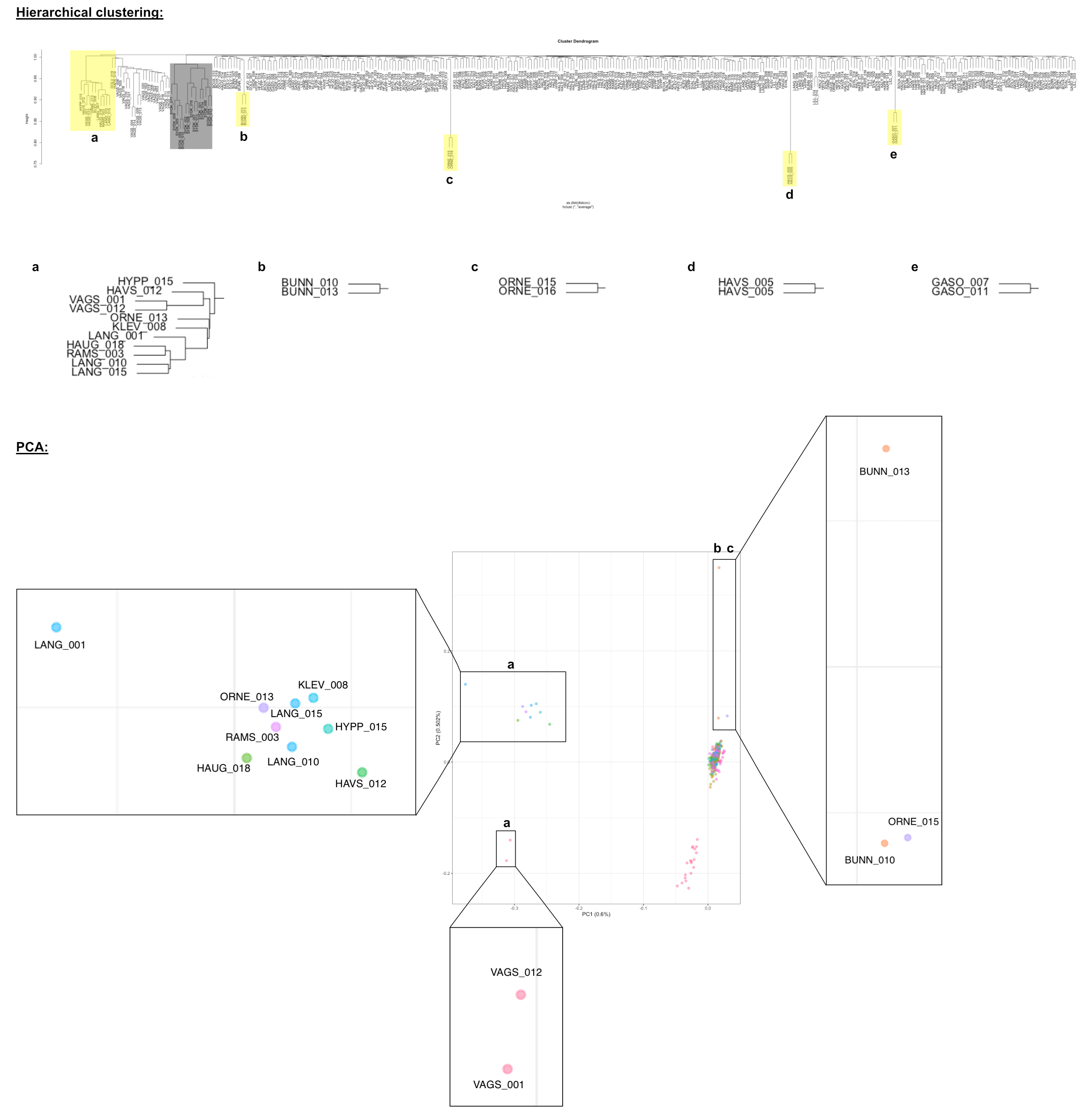

Supplement: Supplementary file 3 — Figure S3. Hierarchical clustering and PCA on a pre‐filtered dataset of flat oyster sequences, including technical replicates and closely related individuals. The yellow rectangles in the hierarchical clustering highlight the sequences that were removed for further analyses. The grey rectangle highlights hatchery‐bred samples that were not included in the study. The group a was removed because of weird clustering, groups b, c, d and e were removed from the dataset because they are composed of closely related individuals. [file EVA-18-e70096-s011.png]

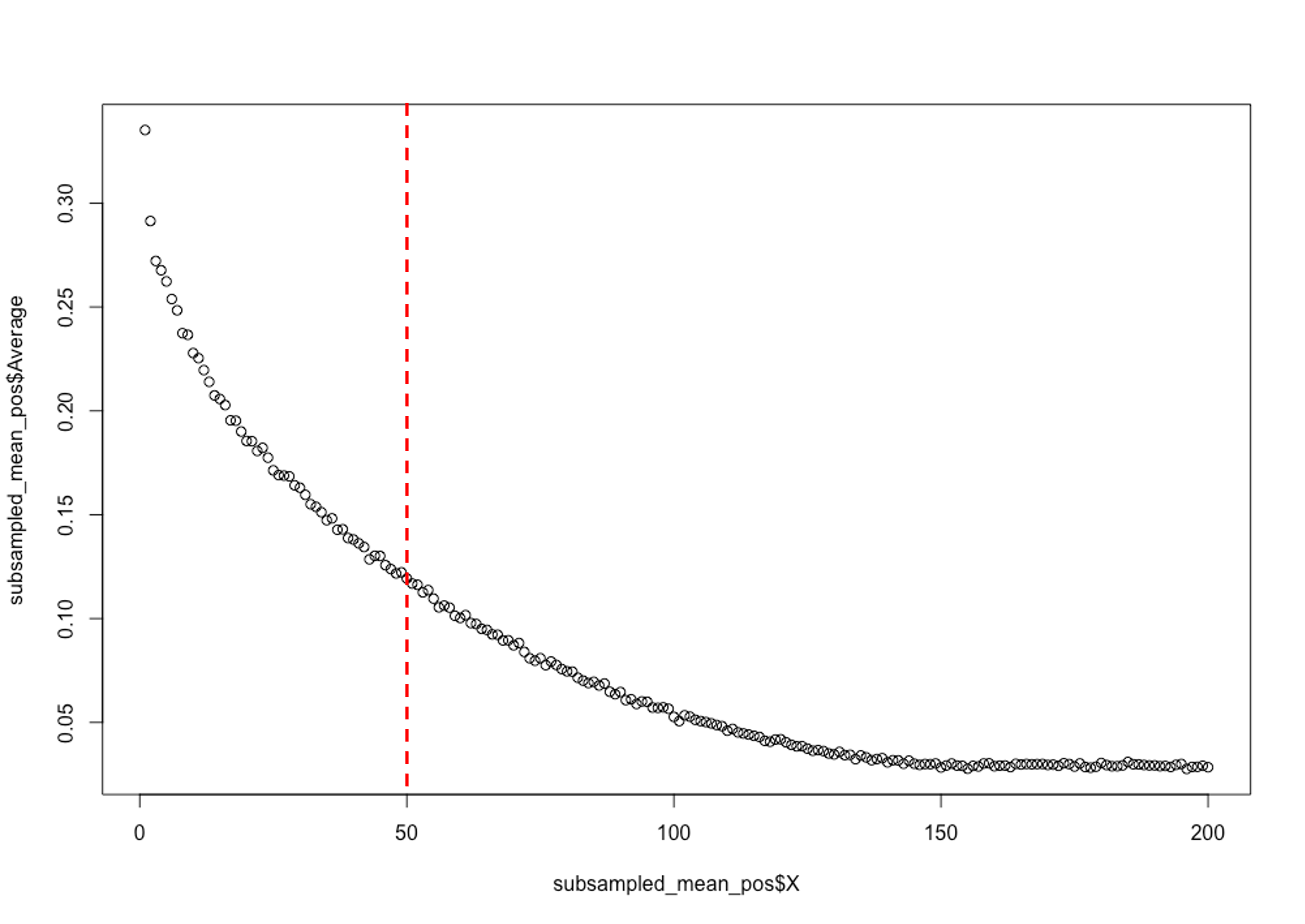

Supplement: Supplementary file 4 — Figure S4. LD‐decay plot along scaffold 4. The red dotted line represents the threshold selected for linkage pruning. [file EVA-18-e70096-s002.png]

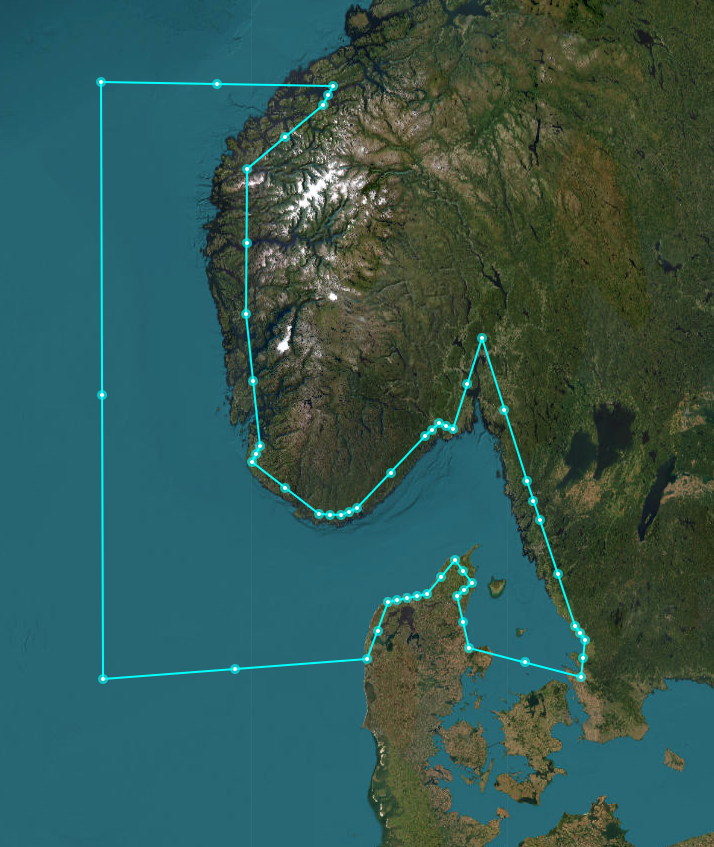

Supplement: Supplementary file 5 — Figure S5. Polygon used for the EEMS analysis. The coordinates were obtained using the Polyline tool available on the Keene State College website https://www.keene.edu/campus/maps/tool/. [file EVA-18-e70096-s010.png]

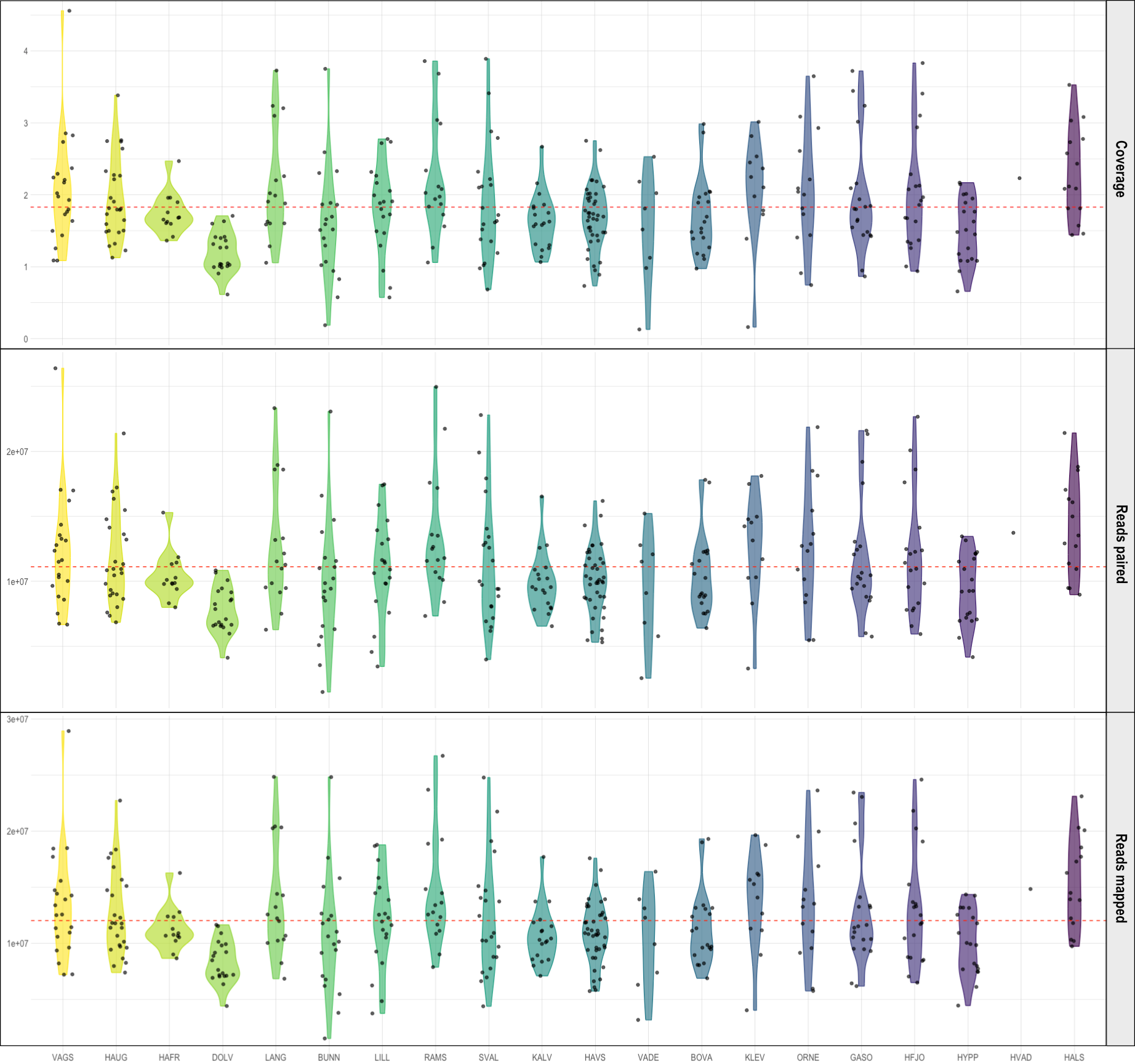

Supplement: Supplementary file 6 — Figure S6. Depth of coverage, number of reads paired and mapped for each sample. One dot represents one individual. [file EVA-18-e70096-s007.png]

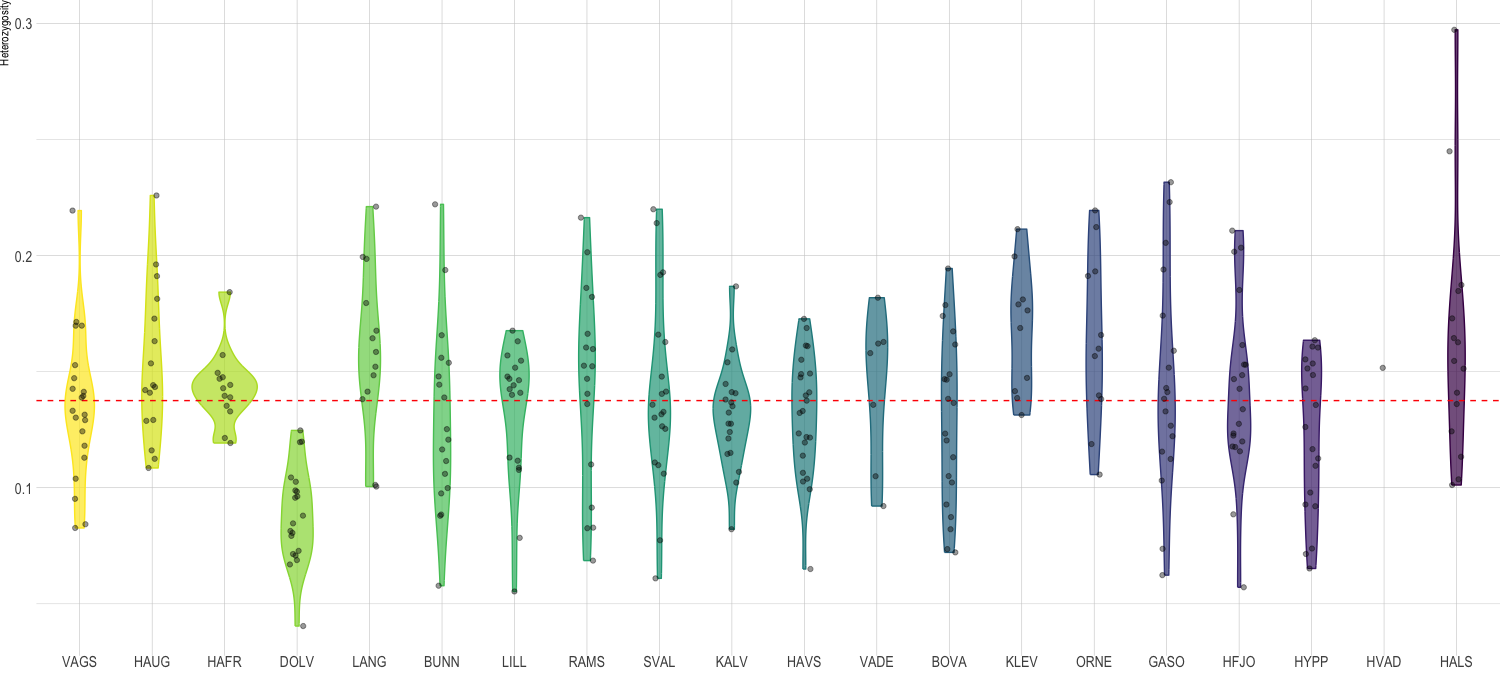

Supplement: Supplementary file 7 — Figure S7. Proportions of heterozygous genotypes in each sample, using Dataset I. The red dotted line corresponds to the average level of heterozygosity calculated for all samples. One dot represents one individual. [file EVA-18-e70096-s003.png]

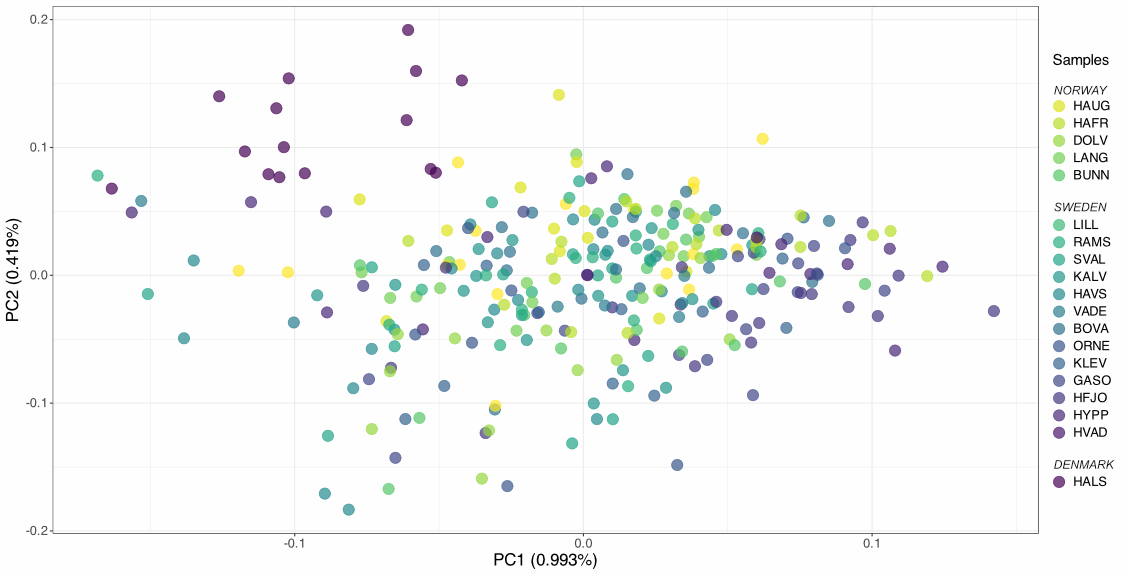

Supplement: Supplementary file 8 — Figure S8. Visualisation of first and second principal components of a principal component analysis (PCA) on 298 flat oysters based on 1,840,068 SNPs (Dataset IV). Individuals are represented by dots coloured by their sample of origin. The percentage of variation explained by each principal component is specified on each axis. [file EVA-18-e70096-s014.png]

A.

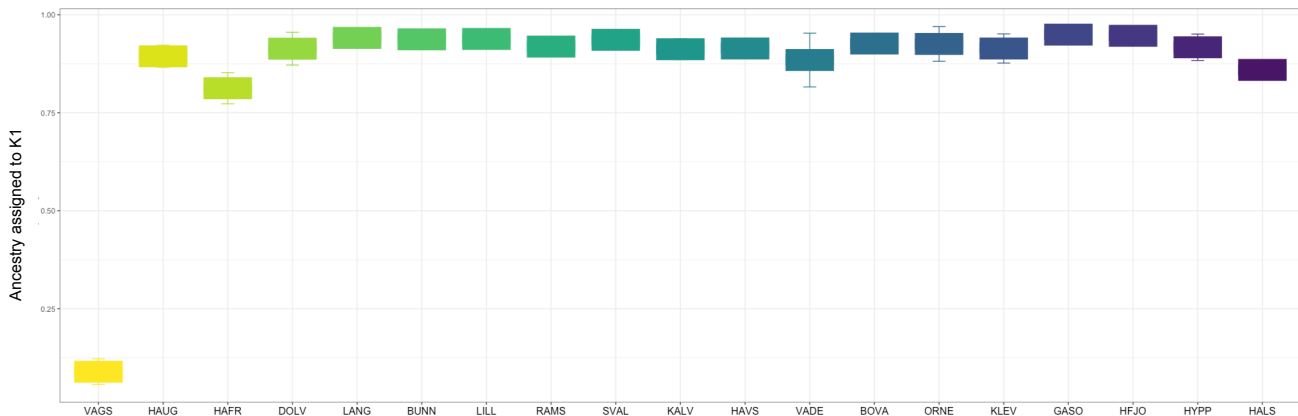

B.

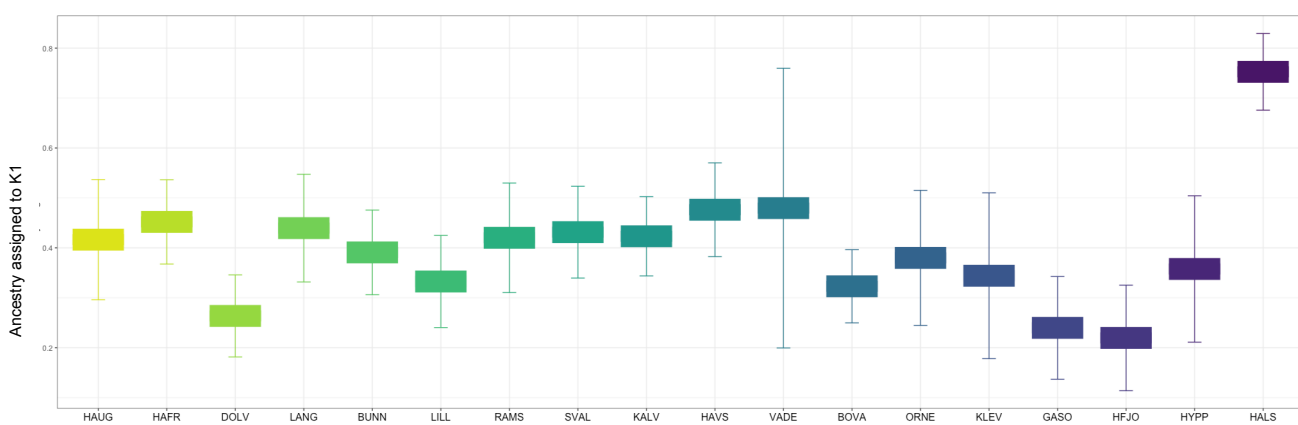

Supplement: Supplementary file 9 — Figure S9. Proportion of each sample assigned to K1, from the PCAngsd admixture analysis. (A) Using Dataset III, that is, 20 sampling locations and 319 flat oysters; based on 1,840,068 SNPs. (B) Using Dataset IV, that is, 19 sampling locations and 298 flat oysters; based on 1,840,068 SNPs. [file EVA-18-e70096-s012.pdf]

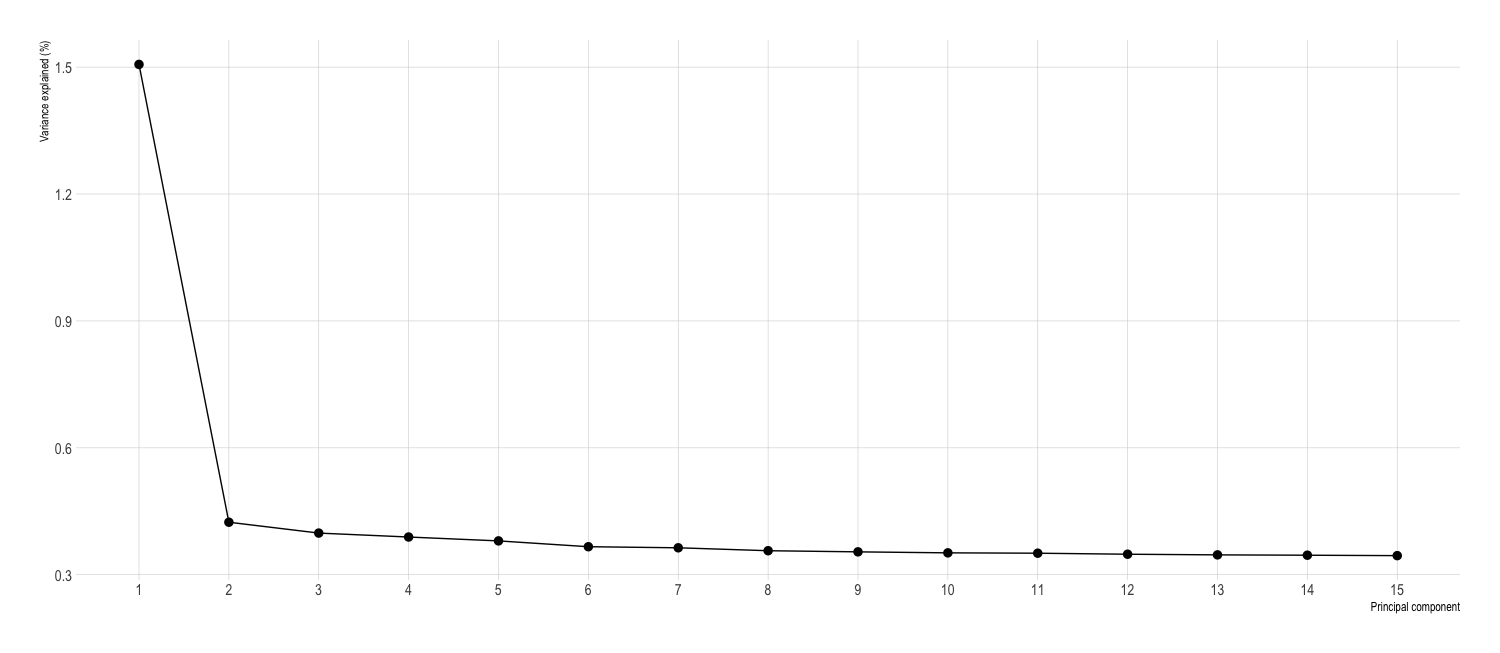

Supplement: Supplementary file 10 — Figure S10. Scree plot, percentage of variance explained by each principal component, based on the PCAngsd covariance matrix computed for Dataset III. [file EVA-18-e70096-s006.png]

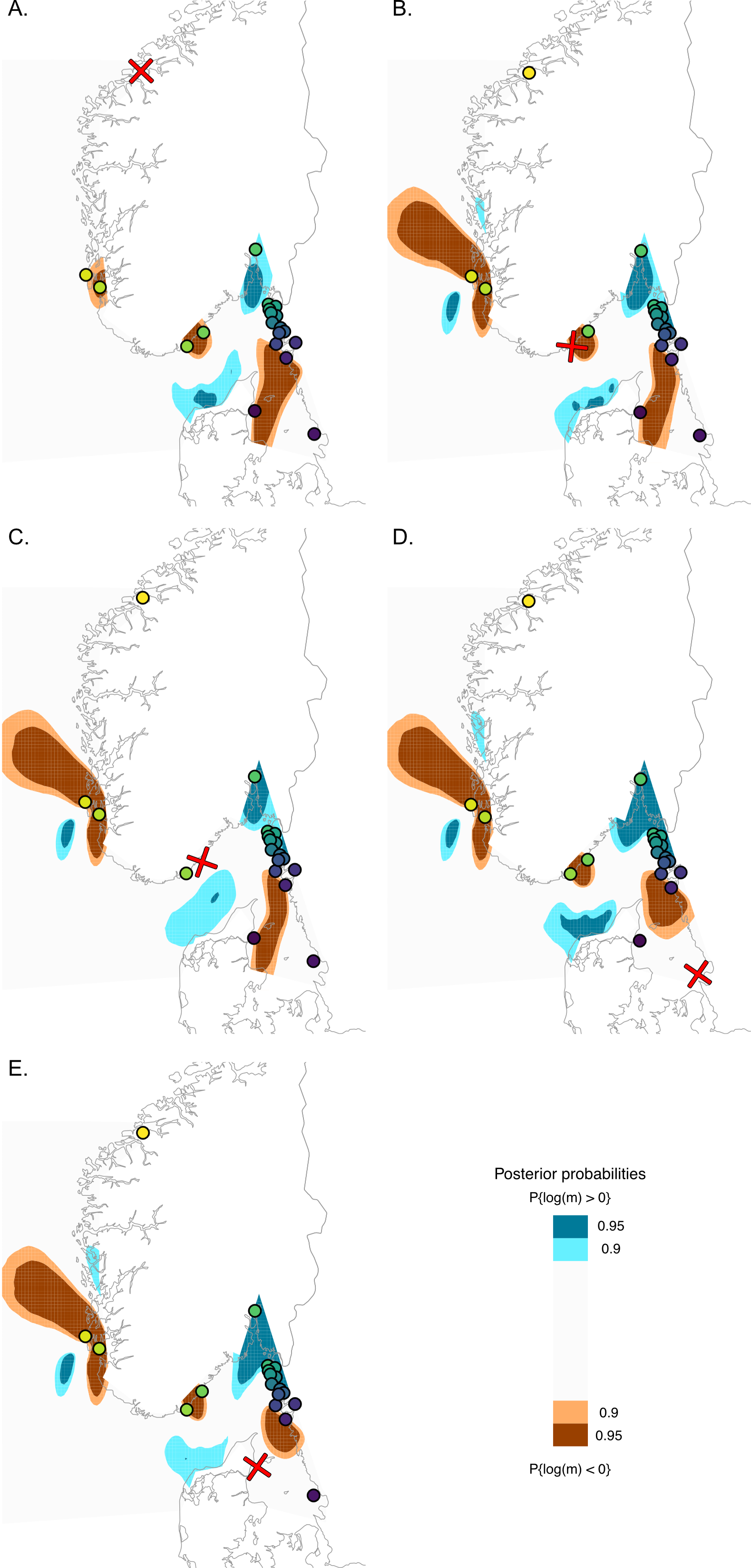

Supplement: Supplementary file 11 — Figure S11. Map showing the effective migration surface, output from the EEMS analysis, after removal of a sample from Dataset III (1,840,068 SNPs). The following samples were removed for each map: (A) “VAGS”; (B) “DOLV”; (C) “LANG”; (D) “HVAD”; (E) “HALS”. Geographic barriers are shown in brown, and regions with high migrations are shown in blue. Coloured dots represent the samples included in the analysis. [file EVA-18-e70096-s017.png]

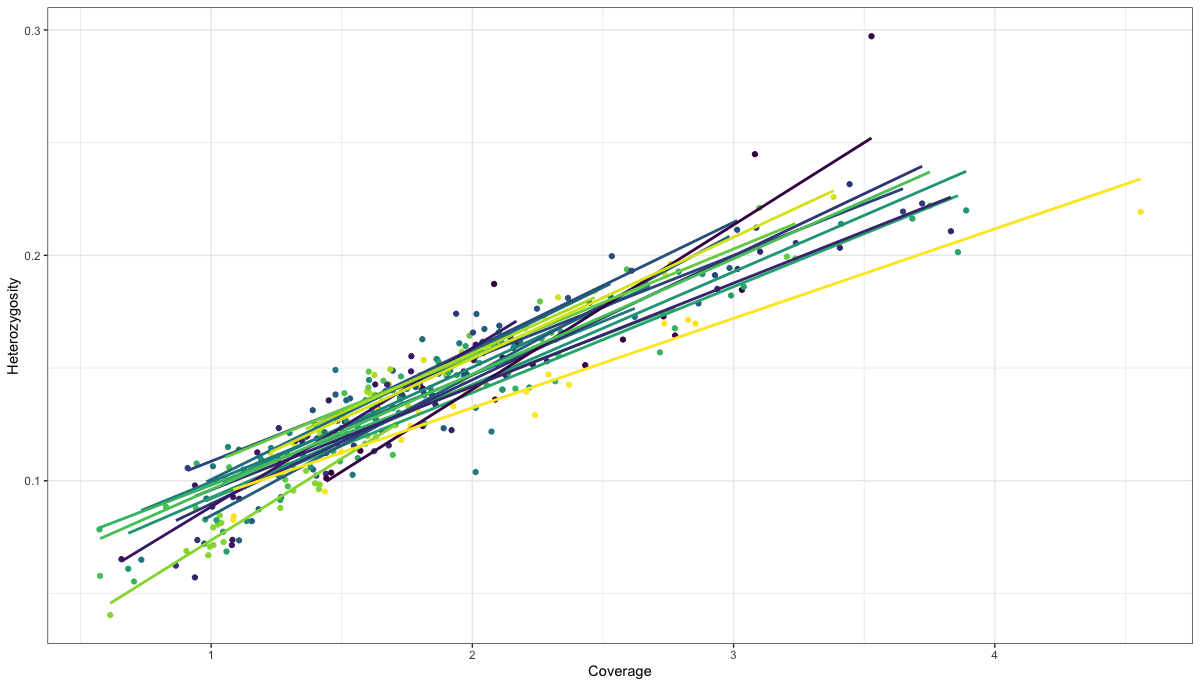

Supplement: Supplementary file 13 — Figure S13. Linear regression model showing the relationship between heterozygosity and coverage for each individual included in Dataset I. Each dot is one individual; the colour represents the sample of origin. [file EVA-18-e70096-s013.png]

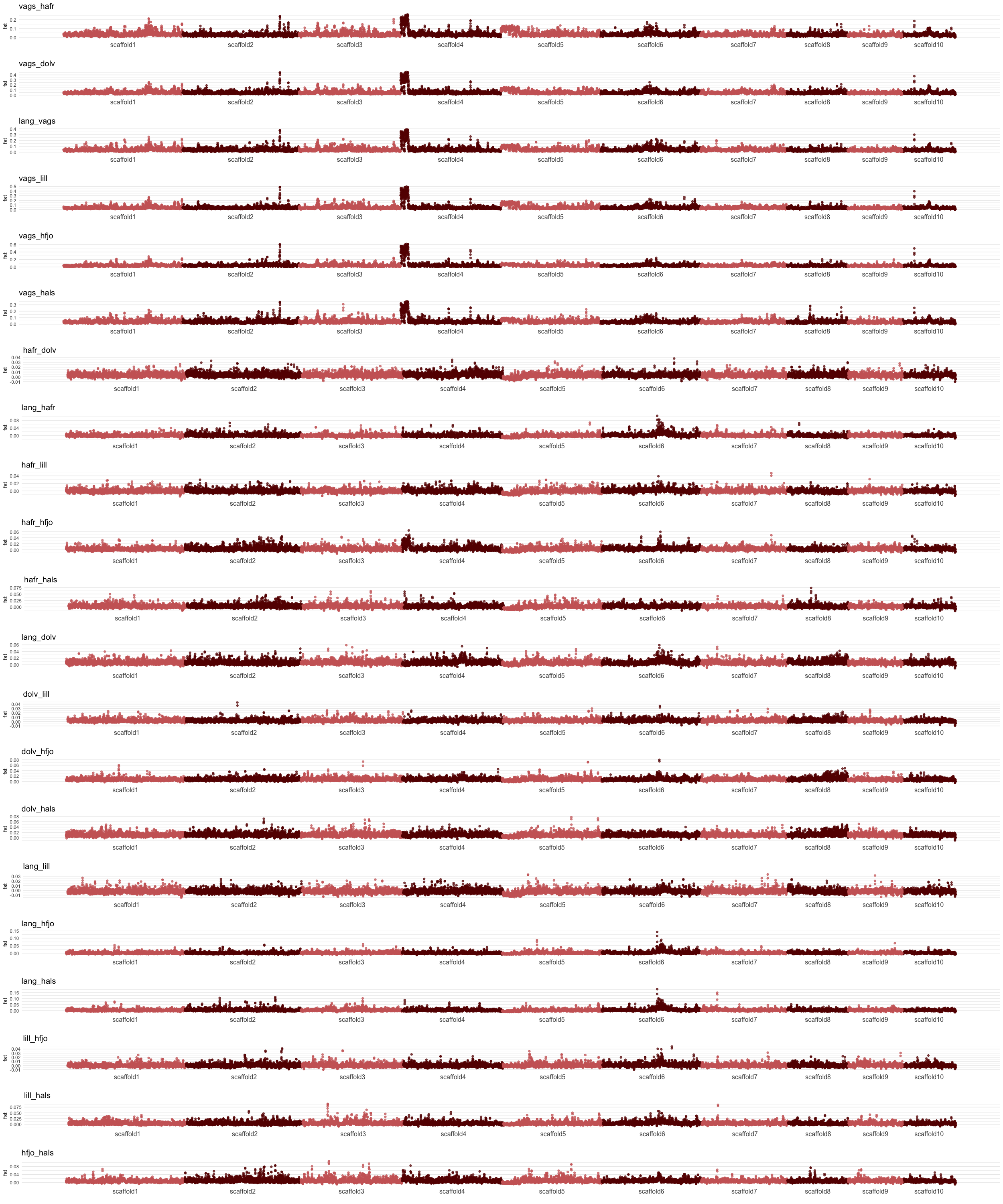

Supplement: Supplementary file 14 — Figure S14. Pairwise Fst with a window size of 100 kb and a step of 50 kb among samples of flat oysters, using Dataset V. The 10 scaffolds are highlighted with different colours. [file EVA-18-e70096-s015.png]

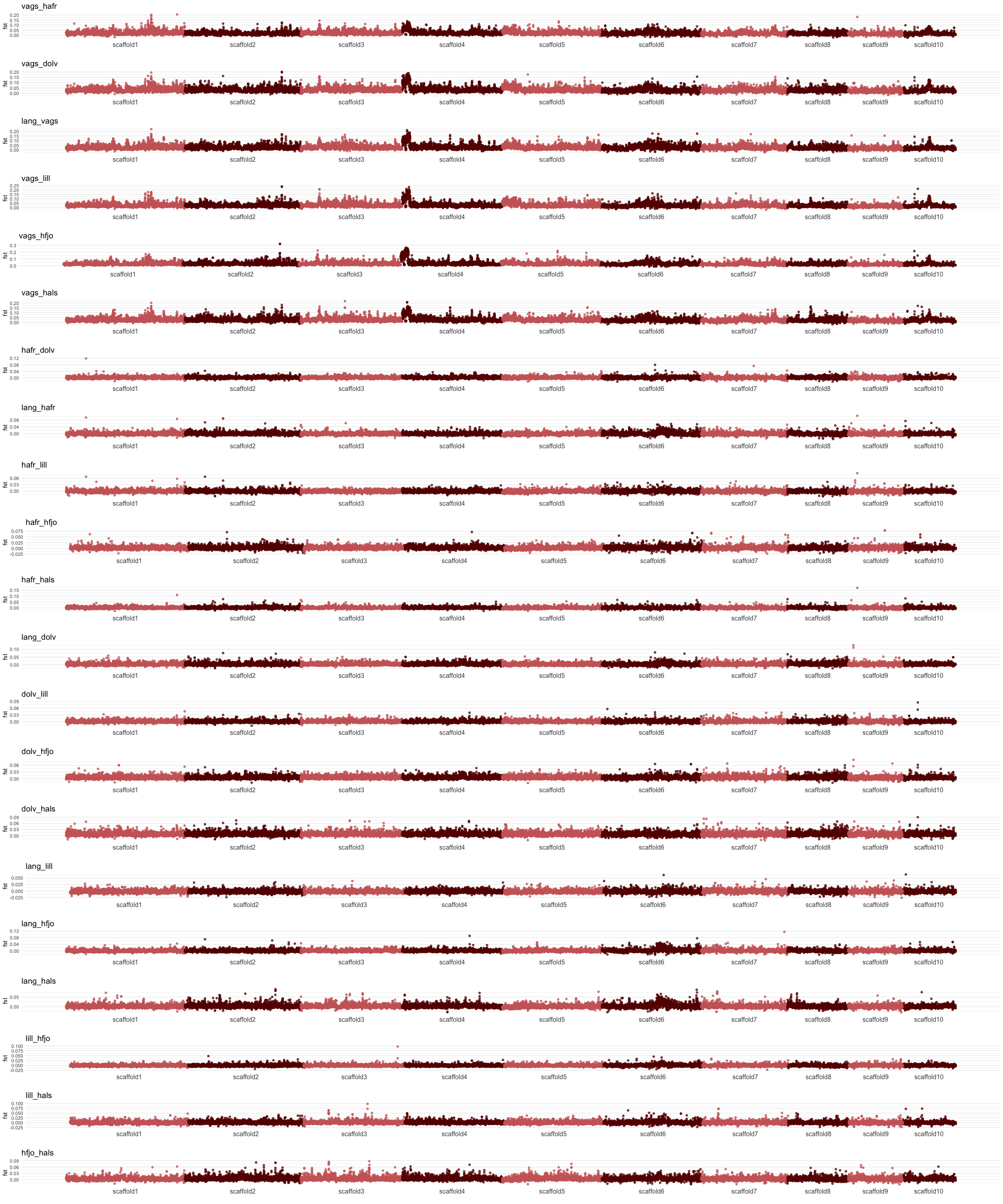

Supplement: Supplementary file 15 — Figure S15. Pairwise Fst with a window size of 100 kb and a step of 50 kb among samples of flat oysters, using Dataset VI. The 10 scaffolds are highlighted with different colours. [file EVA-18-e70096-s005.png]

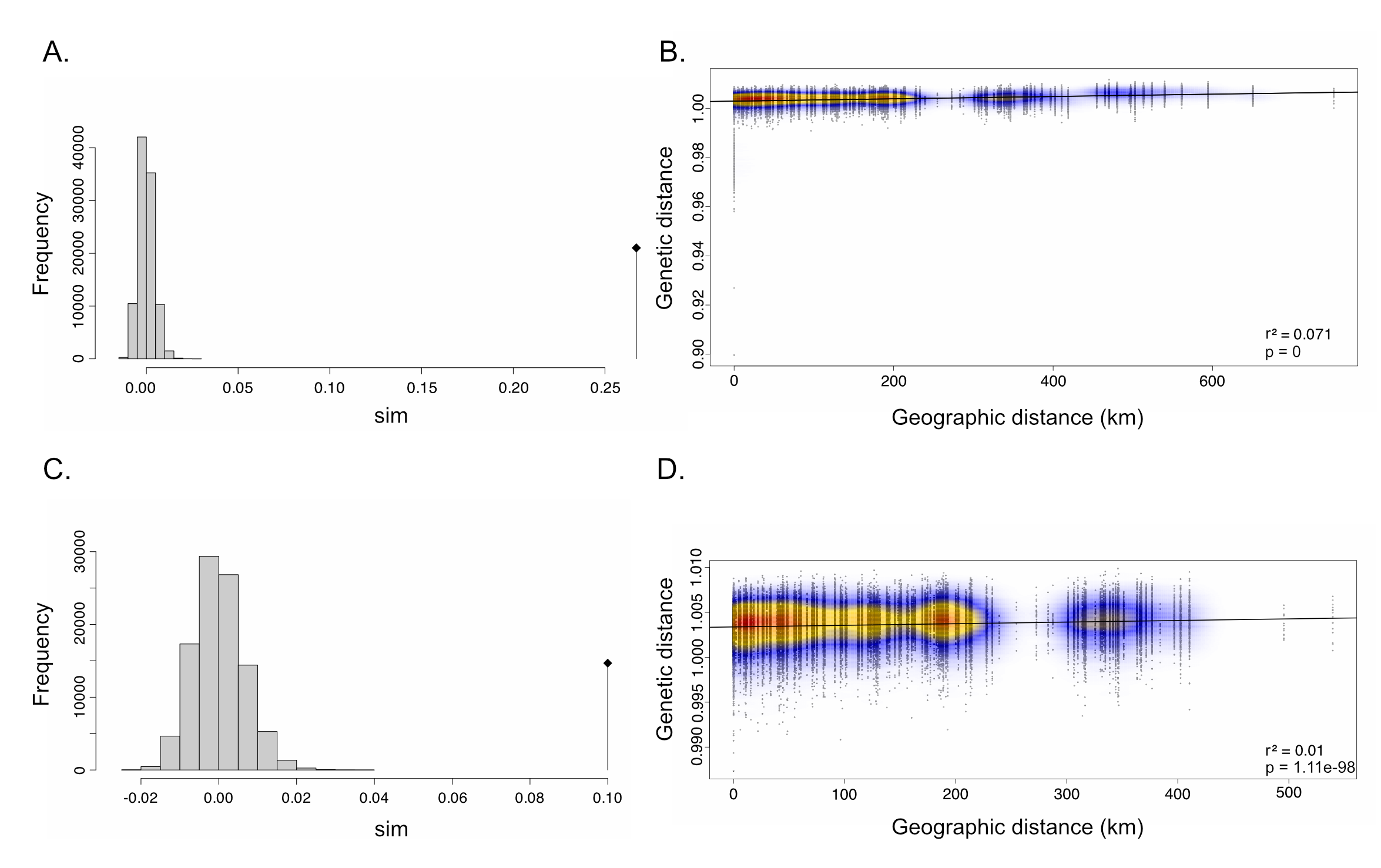

Supplement: Supplementary file 17 — Figure S17. Analyses of correlation between genetic and geographic data for the entire dataset of flat oysters included in this study (Dataset III; A, B panels) and for the dataset excluding the VAGS sample (Dataset IV; C, D panels). The genetic dataset is the covariance matrix output from ANGSD, and geographic data has been calculated as the shortest distance between two samples. (A, C) Histograms showing the distribution of 100,000 simulated Mantel correlations, the vertical line shows the observed correlation between the matrices of genetic and geographic data. For both datasets, p < 0.00001. (B, D) linear regression between geographic and genetic distance. Colours show the change in density of data points. (B) Slope = 4.727e‐06, r 2 = 0.071, p < 0.001, D: Slope = 1.795e‐06, r 2 = 0.01, p < 0.001. [file EVA-18-e70096-s009.png]
